# Highly Elastic and Conductive Lamellar Wood Sponge via Cell Wall Reconfiguration Toward Smart Multifunctional Applications

**DOI:** 10.1007/s40820-025-02016-4

**Published:** 2026-01-05

**Authors:** Xin-jian Dai, Xin Wang, Ji-hang Hu, Pan Jiang, Xiao-qing Wang

**Affiliations:** Research Institute of Wood Industry, Chinese Academy of Forestry, Xiangshan Road, Haidian District, Beijing, 100091 People’s Republic of China

**Keywords:** Cell wall reconfiguration, Wood sponge, Electromagnetic interference shielding, Thermal management, Pressure sensing

## Abstract

Three-dimensional porous foams and aerogels with high compressibility and elasticity hold great promise for applications in pressure sensing, electromagnetic interference (EMI) shielding, and thermal insulation. However, their widespread application is often hindered by compromised structural stability and inadequate fatigue resistance under repeated compression. Herein, a sustainable “top-down” cell wall reconfiguration strategy is proposed to fabricate highly elastic, fatigue-resistant, and electrically conductive lamellar wood sponge from natural balsa wood. This strategy involves the conversion of the intrinsic cellular structure of wood into an arch-shaped lamellar architecture reinforced by chemical cross-linking, followed by coating the lamellar scaffold with conductive polypyrrole (PPy) via in situ polymerization. The resulting PPy-coated cross-linked wood sponge (CWS@PPy) demonstrates reversible compressibility, excellent fatigue resistance (∼3.5% plastic deformation after 10,000 cycles at 40% strain). The strain-induced conductivity changes in CWS@PPy enable tunable EMI shielding effectiveness under cyclic compression and also facilities high-sensitivity pressure sensing (0.72 kPa^−1^). Additionally, CWS@PPy exhibits a low through-plane thermal conductivity of 0.037 W m^−1^ K^−1^, which can be dynamically tuned for adaptive thermal management. The proposed mechanically robust and conductive wood sponge provides a versatile and sustainable platform for next-generation smart devices.

**Supplementary Information:**

The online version contains supplementary material available at 10.1007/s40820-025-02016-4.

## Highlights

- Highly elastic, fatigue-resistant, and conductive lamellar wood sponges are developed via a cell wall reconfiguration strategy.
- The strain-induced conductivity changes in lamellar wood sponge enable tunable electromagnetic interference shielding effectiveness and high-sensitivity pressure sensing (0.72 kPa^−1^).
- The wood sponge exhibits a low through-plane thermal conductivity of 0.037 W m^−1^ K^−1^, which is compression-tunable for smart thermal management.

**Supplementary Information:**

The online version contains supplementary material available at 10.1007/s40820-025-02016-4.

## Graphical abstract

**Figure:**
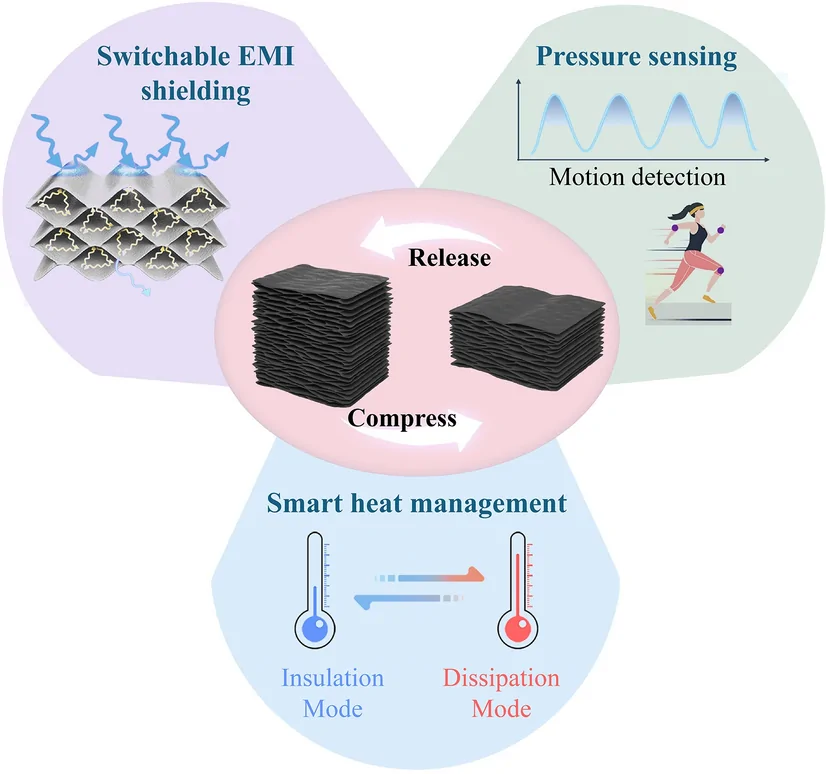

## Introduction

Three-dimensional (3D) compressible and elastic porous materials (CEMs) show great potential for a wide range of applications, including thermal insulation [1–3], oil/water separation [4–6], pressure sensing [7], and electromagnetic interference (EMI) shielding [8, 9]. The versatility of CEMs can be attributed to their ability to undergo reversible microstructural change during compression and subsequent rebound. However, maintaining the structural integrity and mechanical resilience of these materials while minimizing energy dissipation during repeated compression remains a challenge, which significantly impacts the reliability of CEMs in practical applications. Therefore, rational design of the microstructure of CEMs is crucial for achieving high compressibility, superior elasticity, and excellent fatigue resistance [10]. As a typical structure enabling high compressive elasticity, the arch-shaped lamellar structure has attracted great interest in the structural design of high-performance CEMs [11]. From a mechanical perspective, the lamellar structure can accommodate large compression by bending and straightening the arch-shaped lamellas and revert to its original shape immediately upon the release of stress, while preserving the structural integrity [12, 13]. In fact, such arch-shaped spring-like structures are widely used in various vehicles for shock absorption and shaft support [14].

Inspired by the arch-shaped spring-like structures, a variety of CEMs with a similar lamellar architecture have been synthesized from various building blocks including carbon nanotubes (CNTs) [15], SiO_2_ nanofibers [16–18], graphene oxide (GO) [19–21], MXene nanosheets [22] using diverse bottom-up assembly strategies, such as self-assembly [23–25], directional freezing [26, 27], and 3D printing [28, 29]. Among them, directional freezing stands out as an effective approach for fabricating CEMs with aligned pore channels, in which the ordered ice crystals serve as templates for guiding the assembly of the building blocks into an anisotropic lamellar structure [30, 31]. However, owing to the weak interaction between the pure building blocks, the fabricated CEMs often suffer structural damage and display poor fatigue resistance when subjected to prolonged cyclic compression [11, 32]. To address this issue, polymers and amorphous carbon have been incorporated to reinforce the junctions in the scaffold, thereby enhancing the mechanical stability of the lamellar structure [12, 33]. For example, the arch-shaped chitosan/GO composite aerogel, fabricated using a bidirectional freezing method followed by thermal annealing, can withstand over 25,000 cyclic compressions at a strain of 50% [12]. Despite the versatility of the directional freezing technique to fabricate anisotropic lamellar CEMs, it remains a challenge to achieve a long-range lamellar structure with consistent alignments and pore sizes due to the difficulty in maintaining the low temperature necessary for directional ice growth. Moreover, the complex procedures and high energy consumption involved in the bottom-up assembly processes may hinder their widespread applications in terms of fabrication efficiency, cost-effectiveness, and scalability. Therefore, developing a facile and inexpensive approach for the fabrication of anisotropic lamellar CEMs with consistent pore alignments is highly desirable yet remains challenging.

**Fig. 1:**
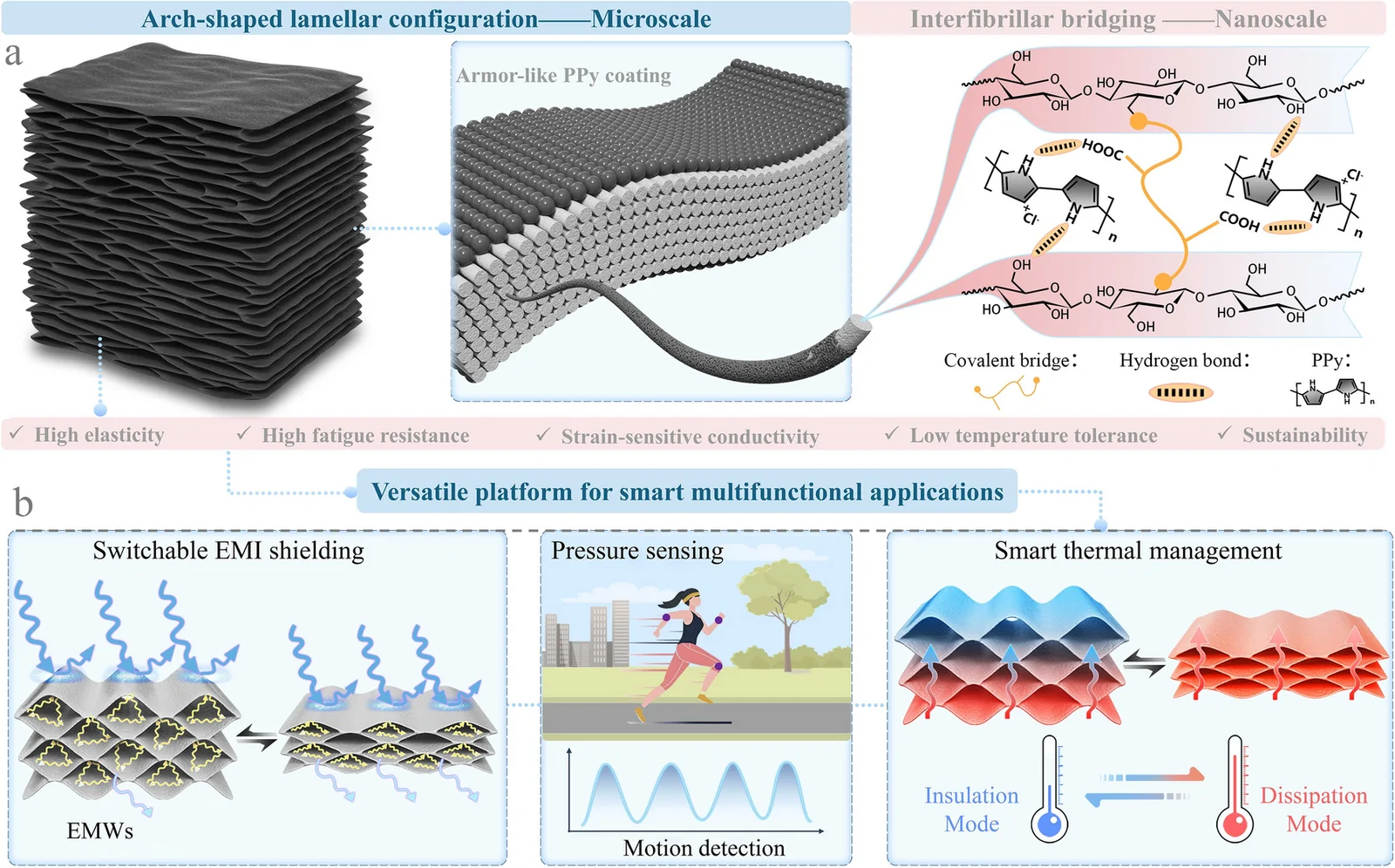
Schematic illustration of **a** highly elastic and **b** conductive lamellar wood sponge via cell wall reconfiguration for smart multifunctional applications

Herein, we proposed a sustainable “top-down” cell wall reconfiguration strategy to fabricate highly elastic, fatigue-resistant, and electrically conductive lamellar wood sponges directly from natural balsa wood (Fig. 1a). This strategy involves the conversion of the cellular structure of wood into an arch-shaped lamellar structure at the microscale to achieve high compressibility, and the reinforcement of the interfibrillar connection in the lamella by chemical cross-linking at the nanoscale to ensure high elasticity and fatigue resistance (Fig. S1). A polypyrrole (PPy) coating was subsequently applied on the lamella scaffold via in situ polymerization to endow the cross-linked wood sponge (CWS) with favorable electrical conductivity. Benefiting from the cell wall reconfiguration strategy, the resultant PPy-coated CWS (CWS@PPy) demonstrates reversible compressibility and excellent fatigue resistance. Interestingly, due to the compression-induced microstructural changes and the associated variations in electrical conduction pathway, the electrical conductivity of CWS@PPy can be dynamically regulated by varying the compressive strain. This strain-sensitive electrical conductivity of CWS@PPy facilitates tunable EMI shielding performance, allowing for a reversible switch between high and low shielding states, and enables high sensitivity (0.72 kPa^−1^) in pressure sensing while maintaining desirable working stability (Fig. 1b). Moreover, due to the lamellar structure with aligned pore channels, CWS@PPy delivers a low thermal conductivity of 0.037 W m^−1^ K^−1^ perpendicular to the lamella direction, which can be dynamically tuned through compression, allowing for tailored thermal management. This work offers an innovative top-down strategy for fabricating anisotropic lamellar CEMs with high compressive elasticity, excellent fatigue resistance, and tunable conductivity for smart multifunctional applications.

## Experimental Section

### Materials

Balsa wood (*Ochroma pyramidale*) with a density of ~ 100 mg cm^−3^ was used as the raw material. Sodium chlorite (NaClO_2_), sodium hydroxide (NaOH), hydrochloric acid (HCl), 1,2,3,4-butanetetracarboxylic acid (BTCA), sodium hypophosphite (SHP), and acetic acid were purchased from Shanghai Aladdin Chemistry Co. Ltd. (China). Iron chloride hexahydrate (FeCl_3_·6H_2_O), pyrrole, ethanol were purchased from Shanghai Macklin Biochemical Technology Co., Ltd. (China).

### Preparation of CWS@PPy

CWS was prepared by chemically cross-linking the wood sponge using BTCA according to our previously reported method. Briefly, the natural balsa wood cubes were delignified using a 2 wt% NaClO_2_ aqueous solution with a pH of 4.6 at 80 °C for 20 h. The delignified wood samples were then treated with an 8 wt% NaOH solution at 80 °C for 8 h to remove hemicelluloses. The wood-derived cellulose scaffolds were thoroughly rinsed with deionized (DI) water and then immersed in 500 mL of aqueous solutions containing BTCA (7.5 g, 0.032 mol) and SHP (3.75 g, 0.043 mol) for 24 h. After that, the wet wood gels were freeze-dried to achieve wood sponges, which were subsequently heated at 160 °C for 10 min to initiate the esterification reaction in the dry state, resulting in the formation of CWS. The CWS@PPy was prepared by in situ polymerization of pyrrole monomer on the CWS scaffold. Firstly, CWS was immersed in a 0.3 M FeCl_3_ solution containing 0.2 M HCl for 6 h, enabling full chelation of Fe^3+^ with the functional groups on the cellulosic scaffold. Subsequently, the FeCl_3_ solution containing CWS was placed in a cryostat at 0 °C for 1 h of pre-cooling, and then, a 300 mL ethanol solution containing 0.3 M pyrrole was added every 3 min (60 mL each time) to the continuously stirred ferric ion solution for oxidative polymerization. After a certain time of reaction (1/3, 1/2, 1, 2, and 3 h), the samples were taken out and rinsed with deionized water to remove residual chemicals, and then, the samples were air-dried to yield CWS@PPy.

### Characterization

Scanning electron microscopy (SEM, S-4800) and transmission electron microscopy (TEM, JEM-F200) were used to observe the microstructure of the samples. The chemical compositions of samples were analyzed by the Fourier-transform infrared spectroscopy (FT-IR, Nicolet 6700), Raman spectroscopy (LabRAM, Horiba Jobin Yvon), X-ray photoelectron spectroscopy (XPS, ESCALAB 250Xi), and energy-dispersive spectrometer (EDS, IXRF model 550i) attached to the SEM. Compressive mechanical performances were evaluated by applying a constant displacement rate of 5 mm min^−1^ through an universal testing apparatus (MTS Exceed E43). Dynamic mechanical analysis (DMA) was performed by a TA-Q800 instrument over a temperature range of − 70 to 90 °C at a heating rate of 5 °C min^−1^. X-ray diffraction patterns (XRD) were collected using an X-ray diffractometer (Bruker D8 Advance) with a 2° min^−1^ scanning rate. A Keithley 2400 source meter (Tektronix) was employed to measure volume resistance (*R*) of the sample, with the electrical conductivity (*σ*) derived from the following equation:

1 $$\sigma =H/(R\times A)$$

where *H* and *A* are the height and the cross-sectional area of the sample, respectively.

### Electromagnetic Interference Shielding Performance Measurements

The scattering parameters of samples were measured with a vector network analyzer (VNA, CETC AV3672C) based on waveguide method. The measured frequency range of the EM waves falls within the typical X-band (8–12 GHz), which is widely used in network communication for commercial electronic products. The incident EM waves propagate along the layer-stacking direction of CWS@PPy. The shielding effectiveness of samples were calculated based on their scattering parameters (*S11* and *S21*). The absorption coefficient (*A*), reflection coefficient (*R*), and transmission coefficient (*T*), total shielding effectiveness (SE_*T*_), reflection effectiveness (SE_*R*_), and absorption effectiveness (SE_*A*_) of the samples were calculated based on the following formulas:

2 $${R=|S11|}^{2}$$

3 $${T=|S21|}^{2}$$

4 A+R+T=1

5 $${\mathrm{SE}}_{T}=-10\mathrm{log}(T)$$

6 $${\mathrm{SE}}_{R}=-10\mathrm{log}(1-R)$$

7 $${\mathrm{SE}}_{A}=-10\mathrm{log}\left[T/(1-R)\right]$$

The response of EM waves to mechanical compression in CWS@PPy was simulated using COMSOL Multiphysics software by the finite element method (FEM). The simplified models of CWS@PPy (17.57 wt%) with different sizes of 22.9 mm × 10.2 mm × (18.0, 10.8, and 7.2) mm were built. The complex permittivity was set according to the experimental measurements at 10.0 GHz, which represents the middle point of the testing frequency range (8.2–12.4 GHz).

### Thermal Management Performances Measurements

The thermal conductivity of samples was measured at room temperature and ambient pressure using a thermal conductivity meter (Hot Disk TPS2500S, Sweden). The xenon light source (CHF-XM500, Perfectlight Ltd., China) was employed to simulate the sunlight. The surface temperatures of samples were monitored with an infrared thermal imaging camera (TiS75^+^, USA). For the compression-induced thermal response test, a radial section of the CWS@PPy was attached to the surface of a heating platform, while its compressive strain was controlled by a motion machine, and a thermocouple was attached to its upper surface to record the temperature under different strains. The simulations of heat transfer process of CWS@PPy before and after compression were performed by the transient-state FEM using the COMSOL Multiphysics software. The heat transfer in solids module was used as physical field. For the convenience of analysis, the simulation model was simplified to a 2D lamellar model based on the microstructure observed from SEM. The heat source at the bottom of model was loaded with 110 °C heat source.

### Pressure-Sensing Performance Measurements

A custom-built test system was utilized to evaluate the sensing performance of the CWS@PPy sensor. A* z*-axis moving platform (SOFN 7SVM0505) was employed to precisely regulate the compressive ratio of the sensor. The sensor’s resistance changes and current signals under compressive strain were recorded using a Keithley 2400 source meter at a fixed input voltage of 3 V. The sensor’s relative resistance variation (*ΔR*/*R*_*0*_) and sensitivity (*S*) were calculated using the following equations:

8 $$\Delta R/{R}_{0}=({R}_{0}-R)/{R}_{0}$$

9 $$S=(\Delta R/{R}_{0})/\Delta P$$

where *R₀* is the initial resistance, *R* is the instantaneous resistance, and *ΔP* is the relative change in pressure.

## Results and Discussion

### Fabrication and Characterization of the CWS@PPy

**Fig. 2:**
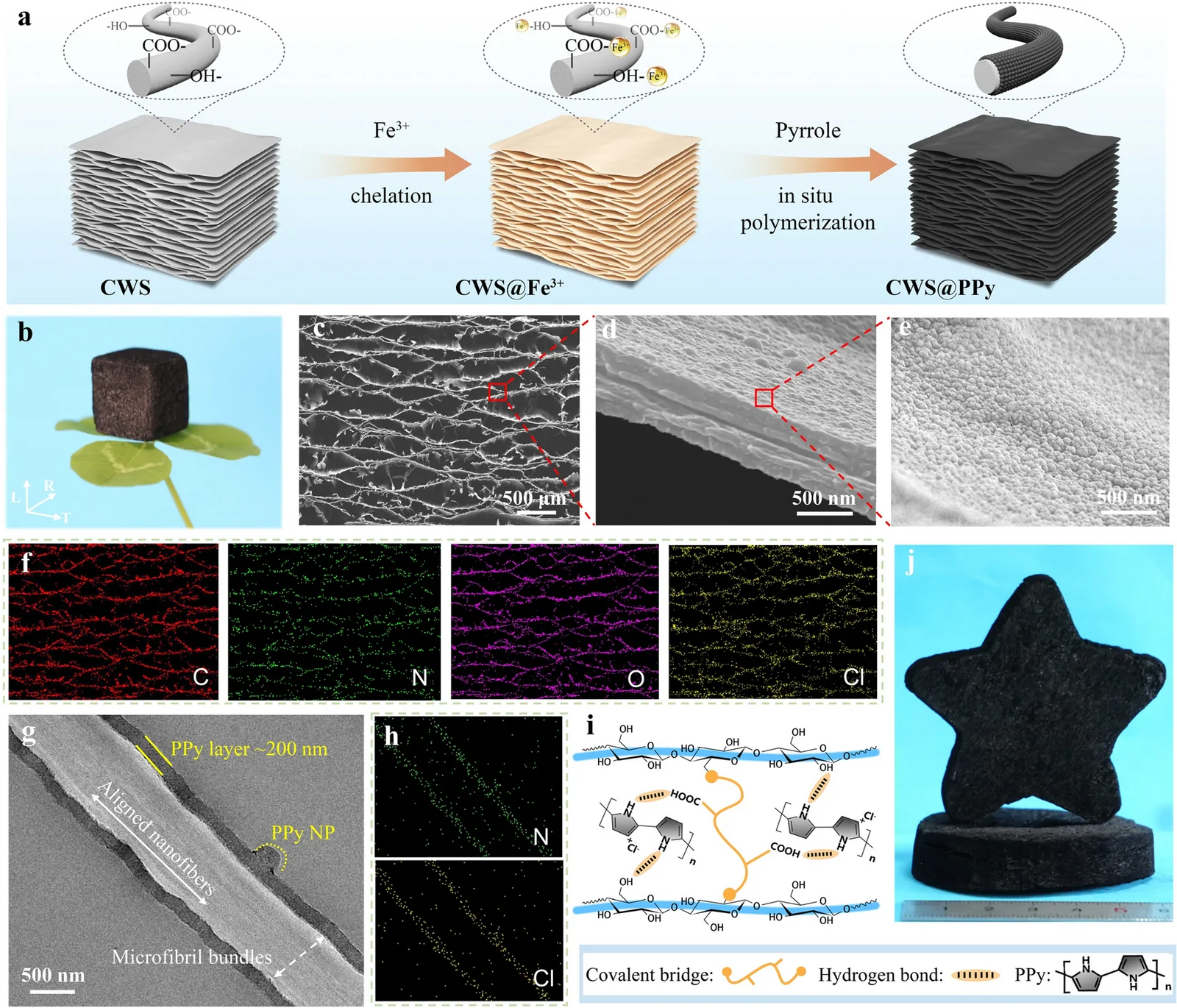
Fabrication and characterization of CWS@PPy. **a** Schematic illustration of the fabrication process of CWS@PPy. **b** Photograph of the lightweight CWS@PPy standing on the leaves of clover. **c** Cross-sectional SEM image showing the lamellar structure. **d–e** SEM images showing the uniform deposition of the PPy nanoparticles on the lamella of CWS scaffold. **f** EDS maps depicting the elemental distribution of C, N, O, and Cl on the lamellar scaffold of CWS@PPy. **g** TEM image and **h** the corresponding EDS mapping images showing the continuous PPy nanocoating on the surface of the microfiber bundle. **i** Schematic illustration of the hydrogen bonding interaction between PPy and the cellulose scaffold. **j** Photograph of CWS@PPy with different sizes and shapes

Figure 2a shows the specific fabrication process of CWS@PPy. The CWS was prepared by chemical treatments to remove lignin and hemicelluloses and break the cell walls of balsa wood, followed by chemically cross-linking the cellulose scaffold with BTCA, resulting in an arch-shaped lamellar structure upon freeze-drying [34]. The successful cross-linking reaction between BTCA and cellulose nanofibrils in CWS can be confirmed by the significant increase in intensities of the ester carbonyl band at 1725 cm^−1^ and the C–O stretching band at 1155 cm^−1^ in the FT-IR spectrum of CWS@PPy (Fig. S2). The prepared CWS exhibited a faint yellow appearance featuring an arch-shaped lamellar structure with interlayer spacing ranging from ~ 50 to 400 µm (Fig. S3). Notably, the interfibrillar cross-linking with BTCA not only enhanced the water resistance of CWS but also facilitated the incorporation of carboxyl groups with high electronegativity serving as active sites for chelating with Fe^3+^ ions, as evidenced by the golden yellow color of the CWS after the infiltration of FeCl_3_ solution (Fig. S4). The chemical oxidation of FeCl_3_ facilitated the in situ polymerization of the pyrrole monomers into PPy on the CWS scaffold (Fig. S5) [35]. The as-prepared CWS@PPy displayed a consistent deep black appearance from the inside out, indicating the formation of conductive PPy across the entire lamellar scaffold (Fig. S6). Notably, anionic doping plays a critical role in enhancing the conductivity of PPy given its inherently poor electrical conductivity [36]. FeCl_3_ serves not only as an oxidant but also provides Cl^−^ as a counterion to be integrated into the pyrrole ring, as evidenced by the peaks at 399.8 and 401.3 eV in the N 1* s* XPS spectrum of CWS@PPy, corresponding to –NH^−^ groups and positively charged N^+^, respectively (Fig. S7) [35, 37].

The porous CWS@PPy has a low bulk density of ~ 60 mg cm^−3^ and is sufficiently light to rest on a plant leaf without causing any visible deformation (Fig. 2b). The cross-sectional SEM image clearly shows the well-preserved lamellar scaffold of the pristine CWS after PPy coating (Fig. 2c). The magnified SEM image reveals that the lamellas of CWS were densely and uniformly coated with the PPy nanoparticles, forming an armor-like nanocoating on the cellulosic scaffold (Fig. 2d, e). In contrast, the CWS prepared by dip-coating shows substantial agglomeration and an uneven dispersion of PPy nanoparticles on its skeleton, thereby highlighting the clear advantage of the in situ polymerization approach (Fig. S8). The successful polymerization of pyrrole within the scaffold also can be confirmed by the appearance of the characteristic peaks at 1526 cm^−1^ (C =C stretching vibration of pyrrole ring) and 1268 cm^−1^ (C–N stretching vibration of pyrrole ring) in the FT-IR spectrum of CWS@PPy (Fig. S9) [35]. Raman spectra further verify the presence of PPy with prominent D and G bands at 1358 and 1567 cm^−1^, respectively (Fig. S10) [38]. EDS mapping reveals a homogeneous distribution of N element originated from PPy throughout the lamellar scaffold (Fig. 2f), consistent with the appearance of a new N signal in the XPS and EDS spectra (Figs. S6 and S11), suggesting the uniform PPy coating [35]. Notably, the XRD pattern of CWS@PPy overlaps closely with that of CWS, confirming that the amorphous PPy coating does not disrupt the native cellulose I crystalline structure (Fig. S12). To gain further insight into the nanostructure of the PPy coating and its interaction with the cellulose scaffold of CWS, high-resolution transmission electron microscopy (HR-TEM) was performed on the CWS@PPy sample. The TEM images reveal that the PPy nanoparticles are densely and uniformly anchored on the surface of the microfiber bundles of CWS with an intimate interfacial contact, forming a continuous PPy nanocoating with a thickness of ~ 200 nm (Figs. 2g and S13). The corresponding EDS mapping confirms the uniform distribution of N and Cl across the microfiber bundle surface (Fig. 2h). Notably, the well-aligned cellulose nanofibers within the fiber bundles of CWS can be clearly observed from the TEM images. We assume this intimate interfacial contact presumably arises from the hydrogen bonding between the –OH/–COOH groups on the cellulose scaffold and the –NH groups on PPy (Fig. 2i), as evidenced by the flattened O–H peak at 3420 cm^−1^ in the FT-IR spectrum after PPy coating (Fig. S9) [35, 39].

The continuous conductive PPy coating is expected to create conductive networks on the lamellar scaffold of CWS, thus endowing it with favorable electrical conductivity, which can be readily tuned by changing the PPy loadings (Fig. S14). As the polymerization reaction proceeded, the PPy loading in CWS increased progressively and reached a stable level of ~ 17.57 wt% after 2 h reaction. Correspondingly, the electrical conductivity of CWS@PPy perpendicular to the lamella direction gradually increased up to ~ 0.15 S m^−1^, while prolonged polymerization provided no additional benefits due to the saturation of the internal conductive network (Fig. S15). Interestingly, the arch-shaped lamellar structure of CWS@PPy enables the modulation of electrical conductivity by compression (Fig. S16). As the compressive strain increased from 0 to 80%, CWS@PPy (19.61% PPy loading) achieved a 7.35-fold enhancement in electrical conductivity, which can be attributed to the compression-induced interfacial contact between the adjacent lamellae and the formation of more continuous conductive pathways within the scaffold [40]. The anisotropic lamellar structure of CWS results in significant electrical anisotropy, with the conductivity along the fiber direction being approximately 10 times higher than that across the lamellae (Fig. S17). Notably, the shape and size of CWS@PPy can be readily manipulated by structurally modulating the wood precursors, demonstrating the scalability and versatility of our top-down fabrication process (Fig. 2j).

The adhesion of the PPy nanoparticles to the CWS lamellar skeleton, which is crucial for practical performance, was systematically assessed. The CWS@PPy maintained its structural integrity even after 6 h of ultrasonication, as the PPy nanoparticles remained firmly anchored to the cellulose scaffold without detachment (Fig. S18). Furthermore, the CWS@PPy exhibited exceptional chemical stability, as shown by the unchanged clarity of HCl (pH = 1) and NaOH (pH = 12) solutions following a 2-week immersion, confirming its high resistance to acids, alkalis, and water (Fig. S19). These results collectively affirm the strong nanoparticle-skeleton adhesion.

### Superior Compressive Elasticity and Fatigue Resistance of CWS@PPy

**Fig. 3:**
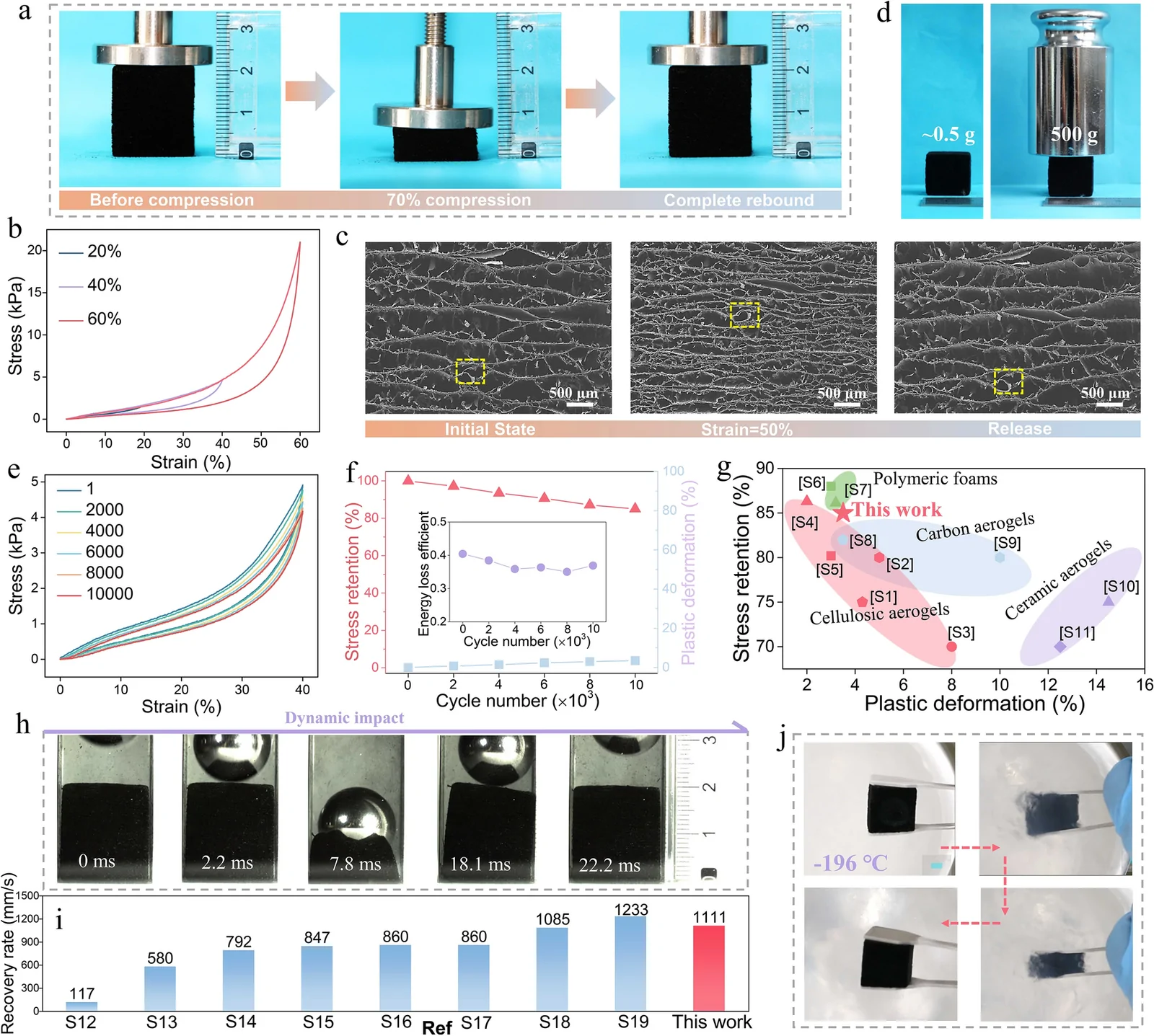
Mechanical properties of CWS@PPy. **a** Photographs of the CWS@PPy showing its reversible compressibility along the layer-stacking direction. **b** Compressive stress–strain curves of the sample at varying strains of 20, 40, and 60%. **c** Microstructural evolution of CWS@PPy during compression and release by in situ SEM observation.** d** Photographs of CWS@PPy bearing a load 1000 times its own weight without deformation along the lamella direction. **e** Compressive stress–strain curves of CWS@PPy at 40% strain for 10,000 cycles. **f** Maximum stress retention, energy loss coefficient, and plastic deformation of CWS@PPy at 40% strain as a function of compression cycles. **g** Comparison of the compressive fatigue resistance between CWS@PPy and previously reported elastic aerogels and foams. **h** High-speed camera images showing the excellent elasticity recovery of  CWS@PPy upon impact with a steel ball. **i** Comparison of the recovery rate of CWS@PPy with those of previously reported 3D compressive and elastic porous materials. **j** Compressive elasticity of CWS@PPy in liquid nitrogen

The mechanical performances of CWS@PPy are crucial for its functional applications. Due to its lamellar structure, the compressive elasticity of CWS@PPy perpendicular to the lamella direction was evaluated using quasi-static uniaxial compression tests. The CWS@PPy can sustain a large compressive strain of up to 70% and fully recover its original height upon stress release, demonstrating high compressibility and elasticity (Fig. 3a). The typical compression stress–strain curve reveals a significant increase in peak stress from ~ 3 to ~ 23 kPa as the compressive strain rises from 20% to 60% (Fig. 3b). Notably, the stress–strain curve at 60% strain reveals three typical deformation stages commonly observed in porous CEMs: a linear elastic stage for *ε* < 10%, attributed to the elastic deformation of the arch-shaped lamellar structure; a plateau stage for 10 < *ε* < 40%, corresponding to the progressive closure of the interlayer spacing; and a sharp rising stage for *ε* > 40%, caused by the densification of the porous lamellar scaffold. Upon unloading, the curve can return to its origin, indicating complete elastic recovery. The in situ SEM observations confirm the reversible deformation of the arch-shaped lamellar structure of CWS@PPy without structural damages during the compression and decompression processes (Fig. 3c). Notably, in contrast with its high compressive elasticity perpendicular to the lamella direction, CWS@PPy can bear a load 1000 times its own weight without deformation along the lamella direction, demonstrating its pronounced mechanical anisotropy (Fig. 3d).

To assess the compressive fatigue resistance of CWS@PPy, we performed cyclic compression tests at an invariant strain of 40% for 10,000 cycles. The resultant stress–strain curves share a similar shape with relatively narrow hysteresis loops, indicating high structural stability (Fig. 3e). After 10,000 cycles, CWS@PPy exhibited only ~ 3.5% plastic deformation and retained over ~ 85% of its maximum stress, demonstrating outstanding compressive fatigue resistance (Fig. 3f). The calculated energy dissipation factor shows a slight decline within the first 4000 cycles and then stabilizes at ~ 0.35 (inset of Fig. 3f), reflecting a small energy loss during cyclic compressions. This mechanical durability can be attributed to the multiscale cell wall reconfiguration strategy, where the arch-shaped lamellar structure can achieve high compressive elasticity and the interfibrillar cross-linking within the lamellar scaffold can effectively suppress the relative slippage of cellulose nanofibers under repeated compression, thereby significantly reducing the energy dissipation and improving its compressive fatigue resistance [34]. The exceptional compressive fatigue resistance of CWS@PPy rivals or even surpasses that of most reported 3D elastic aerogels or foam based on ceramics, synthetic polymers, carbon, and nanocellulose materials (Fig. 3g and Table S1).

Beyond the quasi-static compression test, we further evaluated the dynamic compression performance of CWS@PPy using a typical drop-ball impact test [12, 17, 41]. Specifically, a steel ball (20 g) was freely dropped from a height of 1 m onto a 2-cm-thick CWS@PPy sample (0.5 g) (Fig. S20 and Video S1). High-speed imaging captured the compression and rebound process: The sample reached maximum deformation within ~ 5.6 ms upon impact and completely rebounded the ball in ~ 14 ms, with no visible structural damage (Fig. 3h). The recovery rate of CWS@PPy was calculated to be ~ 1100 mm s^−1^, surpassing most previously reported carbon-, polymer-, and cellulose-based aerogels or foams (Fig. 3i and Table S2), highlighting its excellent mechanical resilience even in dynamic loading conditions.

The mechanical robustness of CWS@PPy was systematically evaluated under various environmental conditions. Dynamic mechanical analysis (DMA) revealed nearly constant viscoelastic properties (storage modulus, loss modulus, and damping ratio) across a broad temperature range of − 70 to 90 °C, indicating exceptional thermo-mechanical stability. (Fig. S21). We also examined the mechanical properties of CWS@PPy at ultra-low temperatures. Impressively, CWS@PPy still retained its compressive elasticity even when exposed to liquid nitrogen (− 196 °C), whereas the conventional synthetic polymer-based PU foam typically lost the original resilience below its glass transition temperature (Figs. 3j and S22; Video S2). Furthermore, we examined the mechanical compressibility of the CWS@PPy in water (Fig. S23). The water-saturated CWS@PPy can be repeatedly compressed up to 50% strain and can fully recover to its original height even after 20 cycles, demonstrating its excellent wet stability. Such high compressive elasticity and exceptional fatigue resistance make CWS@PPy an ideal candidate for various strain-driven functional applications.

### Switchable EMI Shielding Performance of CWS@PPy

**Fig. 4:**
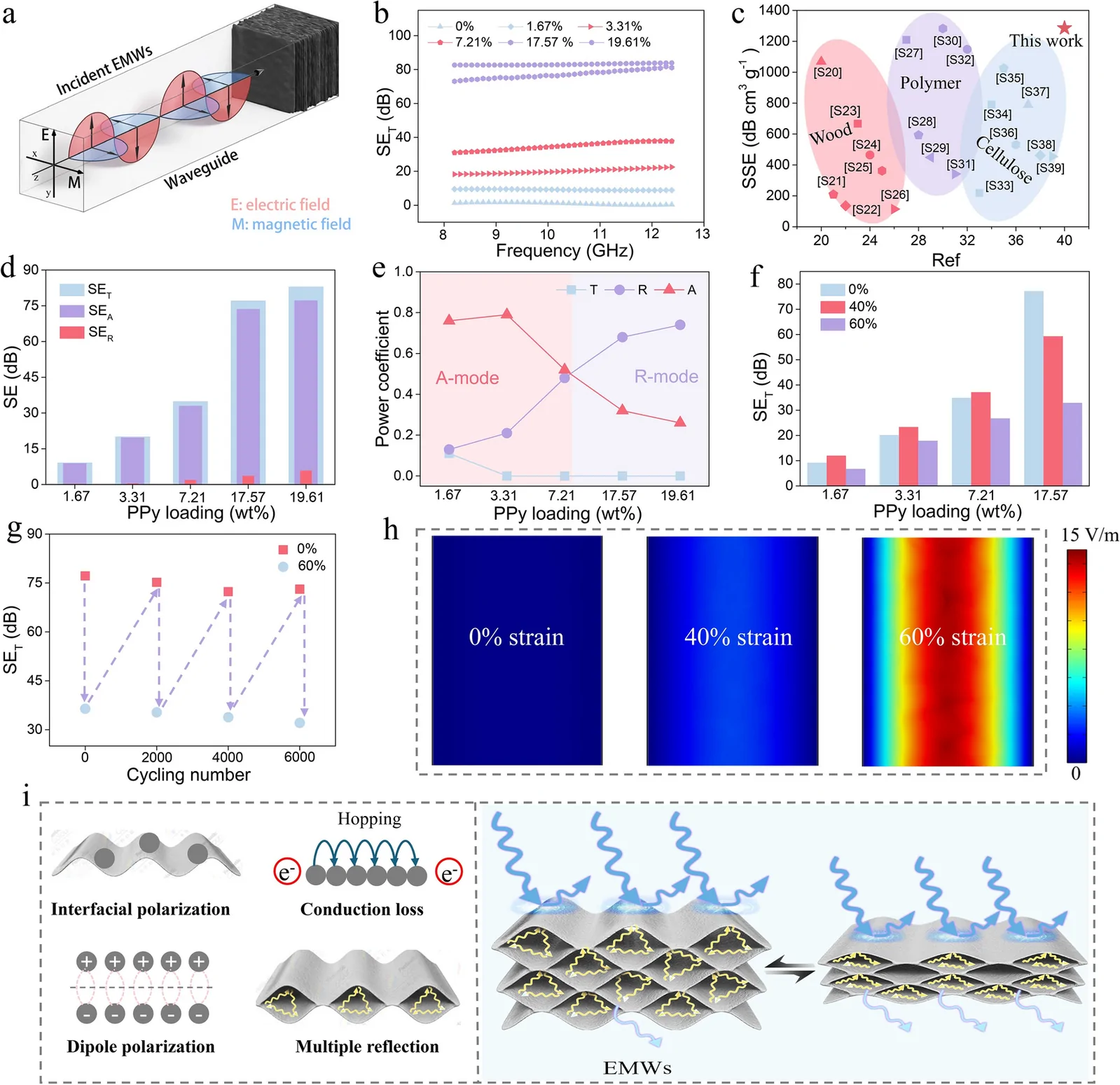
EMI shielding performances of CWS@PPy and the underlying mechanism. **a** Schematic diagram of  CWS@PPy exposed to the incident EM waves in a waveguide cavity. **b** The effect of PPy loading on the SE_T_ of the samples. **c** Comparison of the SSE value of CWS@PPy with those of previously reported aerogels and foams. **d** Average SE_T_, SE_A_, SE_R_ values and **e** power coefficient of the samples with different PPy loadings. **f** SE_T_ of the samples with different PPy loadings under different compressive strains. **g** Reversibly switchable shielding performance of CWS@PPy during cyclic compression. **h** Electric field distribution on the rear surface of the samples with compressive strains of 0, 40, and 60%, respectively. **i** Schematic illustration of the proposed EMI shielding mechanism

The unique arch-shaped lamellar structure and the favorable electrical conductivity of CWS@PPy make it a promising candidate for high-performance EMI shielding materials. Given the critical role of PPy loading in determining the electrical conductivity, we explored the impact of PPy loading on the shielding effectiveness (SE) of CWS@PPy using vector network analysis in the X-band range (8–12 GHz), with the incident electromagnetic (EM) wave propagated perpendicular to the lamella direction (along the Z direction) (Fig. 4a). As shown in Fig. 4b, the SE of CWS@PPy is highly dependent on the PPy loading. The pristine CWS exhibits a negligible SE_T_ value (~ 1 dB) because it cannot conduct electricity, allowing the incident EM wave to readily pass through the entire scaffold and thus causing shielding failure. As the PPy loading increased from 1.67 to 17.57 wt%, the corresponding SE_T_ rose sharply from 9.14 to 77.18 dB. The boosted shielding performance can be attributed to the formation of highly interconnected 3D conductive networks across the cellulose scaffold with a sufficient PPy loading. However, the SE_T_ of CWS@PPy plateaus at 83.09 dB upon reaching a critical PPy loading of 19.61 wt%, indicating the saturation of the conductive pathways. Notably, a twofold greater SE_T_ is observed in the layer-stacking direction than in the fiber direction of CWS, demonstrating significant anisotropy originating from its inherent lamellar structure (Fig. S24). Such a high SE_T_ value far exceeds the 20 dB threshold for commercial EMI shielding applications, affirming its practical potential [35, 42]. Notably, the density of porous 3D EMI shielding materials should be considered while pursuing their high SE values [43]. Benefiting from its low density (~ 60 mg cm^−3^) and high SE_T_, CWS@PPy achieves a high specific shielding effectiveness (SSE, the ratio of SE to density) of 1,286 dB cm^3^ g^−1^, outperforming most reported 3D porous EMI shielding materials (Fig. 4c and Table S3).

We conducted a detailed analysis of the underlying shielding mechanisms of CWS@PPy. Figure 4d depicts the contributions of absorption (SE_A_) and reflection (SE_R_) to the total shielding effectiveness (SE_T_) for samples with different PPy loadings. For all samples, the SE_A_ component accounts for the majority of the total SE_T_, indicating that the entered EM waves are trapped within the porous structure of CWS@PPy, where they are further attenuated and ultimately converted into thermal energy. However, it is insufficient to unveil the shielding mechanism solely by comparing the SE_R_ and SE_A_ values, since the absorption and reflection loss do not represent the actual absorbed and reflected EM energy [44]. To gain deeper insight into the shielding mechanism, we calculated the power coefficients* A* (absorption) and* R* (reflection), which reflect the capability of a material to absorb and reflect EM waves and represent its real EM energy dissipation. For the CWS@PPy with a relatively low PPy loading (< 8 wt%), absorption is dominant (*A* > 0.5 > *R*), while for a high loading (> 8 wt%), reflection becomes prevalent (*R* > 0.5 > *A*) (Fig. 4e), indicating a PPy loading-dependent EMI shielding modes of CWS@PPy. This PPy loading-dependent transition from absorption- to reflection-dominated shielding arises from changes in impedance matching [45]. The CWS@PPy with a low PPy loading exhibits effective impedance matching with free space due to its relatively low electrical conductivity, which facilitates the penetration of the incident EM waves into the porous structure with minimal reflection back into the air. In contrast, the CWS@PPy with a high PPy loading exhibits impedance mismatch due to its high electrical conductivity, resulting in a large portion of EM waves being reflected.

Leveraging its high compressive elasticity and strain-sensitive electrical conductivity, CWS@PPy exhibited strain-tunable EMI shielding behavior. For the convenience of testing, a series of customized plastic molds with varying thickness were used to assemble CWS@PPy for investigating its SE under different strains (Fig. S25). Notably, the SE_T_ of the pure plastic mold is only ~ 1 dB, indicating an insignificant EMI shielding effect (Fig. S26). The variation trend in the SE_T_ of CWS@PPy under compression depends on its PPy loading (Fig. 4f). At low PPy loadings (< 8 wt%), SE_T_ showed an initial increase followed by a subsequent decrease with increasing strain, whereas at a high loading of 17.57 wt%, it monotonically declined from 77.2 to 32.9 dB as the strain increased from 0 to 60%. Notably, the CWS@PPy with a low PPy loading of 3.31 wt% demonstrated an intriguing “off–on” switchable EM response. As the compressive strain increased from 0 to 40%, the SE_T_ rose from 20.1 to 24.2 dB, exceeding the standard commercial shielding requirement (20 dB), representing an “on” state for EMI shielding. However, as the strain continued to increase from 40% to 60%, the SE_T_ decreased from 24.2 to 17.6 dB, falling below the threshold, indicating an “off” state. This “off–on” switchable EMI shielding capability is essential for applications that demand high-precision and intelligent EM protection.

To evaluate the switching stability in EMI shielding, we conducted cyclic compression tests (6000 cycles) on the CWS@PPy with a PPy loading of 17.57 wt% at 60% strain. During the repeated compression/decompression, the SE value consistently and reversibly switched between a high shielding state of ~ 75 dB and a low shielding state of ~ 34 dB without significant attenuation (Fig. 4g). This switching stability stems from the exceptional compressive fatigue resistance of CWS@PPy as well as the robust interfacial bonding between the PPy coating and the CWS scaffold, allowing it to maintain a stable internal conductive network during repeated compression. Finite element simulations of electric field distributions were employed to visualize the strain-induced EM responses of CWS@PPy under compression. As shown in Fig. 4a, the electric field of the EM wave is parallel to the *Y* direction. The distribution of the electric field across the rear surface of the CWS@PPy is illustrated using a 2D color map, which visually shows the variations in electric field intensity (Fig. 4h). When the EM waves penetrated the uncompressed CWS@PPy, the electric field intensity was significantly reduced, indicating that EM waves can hardly pass through the shielding material. By contrast, when the EM waves penetrated the compressed CWS@PPy at a strain of 40 and 60%, the electric field intensity increased significantly, suggesting the penetration of EM waves into the sample to some extent. This simulation aligns well with the experimental results, confirming the strain-dependent modulation of SE.

Building on the above discussion, we proposed the possible mechanisms underlying the exceptional EMI shielding performance of CWS@PPy as well as its strain-dependent switchability of SE (Fig. 4i). The excellent EMI shielding performance (> 70 dB) can be ascribed to the synergistic effects of the following factors: (i) initial reflection of EM waves at the air–material interface due to the impedance mismatch; (ii) ohmic attenuation through electron migration/hopping in the conductive networks [46]; (iii) enhanced interfacial/dipole polarization due to the conductivity difference and Cl^−^ doping; and (iv) multi-reflection/scattering within the arch-shaped lamellar structure [44]. The strain-dependent SE switchability of CWS@PPy is attributed to the competitive effects between its enhanced electrical conductivity and the reduced effective thickness upon compression [47]. At a low PPy loading, an appropriate increase in compressive strain can generate more conductive paths by enhancing the interlayer contact to improve the electrical conductivity, which enhances the impedance mismatch at interfaces and attenuate EM waves by reflection to a certain extent. However, excessive compression significantly reduces the material thickness, and shortens the conductive paths for multiple reflections/scatterings of EM waves within the material. The declined EM wave attenuation due to the reduced material thickness outweighs the increased reflection loss due to the enhanced electrical conductivity induced by compression, ultimately lowering SE_T_. In contrast, at a high PPy loading, the CWS@PPy demonstrates a consistently high electrical conductivity regardless of the applied compressive strain (Fig. S27), making the material thickness the dominant factor controlling its SE and thus giving rise to its monotonic SE decrease with strain. Overall, the high specific EMI SE, broad tunable range, and exceptional switching stability of CWS@PPy make it a highly competitive candidate for tunable EMI shielding applications.

### Pressure-Sensing Performances of the CWS@PPy

**Fig. 5:**
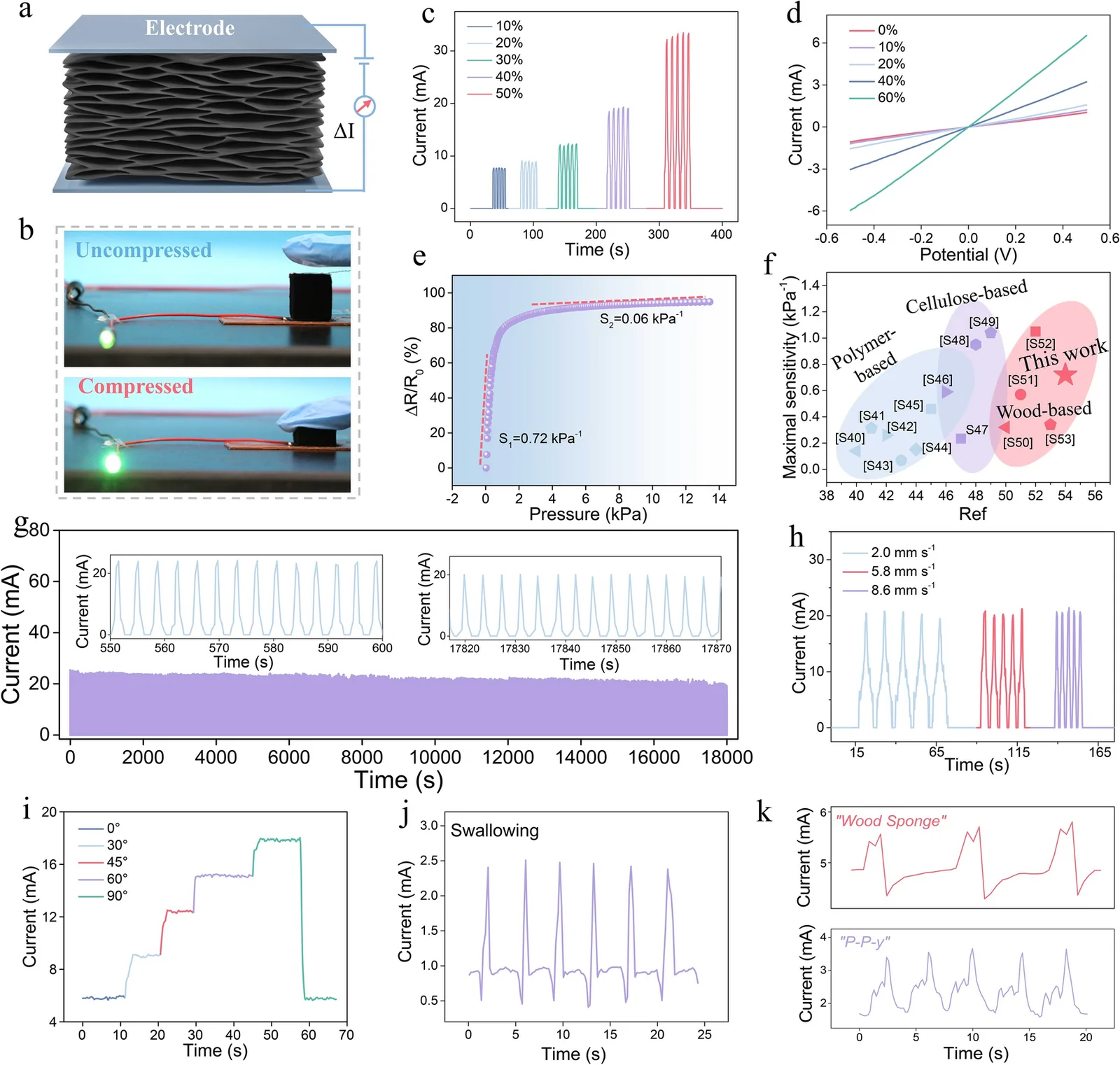
Pressure-sensing performances of CWS@PPy. **a** Schematic of the CWS@PPy sensor structure. **b** Variations in the brightness of an LED lamp connected to a CWS@PPy sensor in a closed circuit before and after compression. **c** Current response of the sensor under different strains. **d** I–V curves of the sensor at different strains. **e** *ΔR/R*_*0*_ of the sensor with the applied pressure.** f** Comparison of the maximum sensitivity of the CWS@PPy sensor with various reported aerogel- and foam-based sensors. **g** Sensing stability of the CWS@PPy sensor over 8000 compression–decompression cycles at 40% strain. **h** Current response of the sensor at 40% strain under varying loading rates. **i–k** Application of CWS@PPy sensor for real-time detecting human motions: **i** finger bending at varying bending angles, **j** swallowing, and **k** pronouncing different words

Due to its strain-sensitive electrical conductivity, CWS@PPy is promising for applications in piezoresistive sensing (Fig. 5a). When connected in series with a LED lamp and powered at 3 V, the LED luminance responded sensitively to the mechanical deformation of CWS@PPy upon compression, qualitatively demonstrating its piezoresistive effect (Fig. 5b). To systematically assess its sensing performances, a pressure sensor was assembled by sandwiching CWS@PPy between two flexible copper electrodes and mounted on a linear motion platform. Under a constant 3 V voltage, the output current increased sharply upon compression and rapidly recovered after release, displaying a strain-dependent trend and excellent reversibility (Fig. 5c). The I–V curves exhibited linear ohmic behavior within the voltage range of − 0.5 to 0.5 V at different strain levels, indicating the consistent electrical resistance of the sensor at varying compression states (Fig. 5d). The slope of the I–V curves increased with increasing strains, reflecting a corresponding decline in electrical resistance, which can be attributed to the enhanced contact between the PPy-coated lamellas and the formation of additional conductive pathways under compression.

The relative resistance change (*ΔR*/*R*_0_) with the applied pressure and strain highlights the high sensitivity of the CWS@PPy sensor (Figs. 5e and S28). A sharp *ΔR/R*_*0*_ increase in the 0–2 kPa (10% strain) range reflects a significant reduction in the internal resistance of CWS@PPy, resulting in a high pressure sensitivity of ~ 0.72 kPa^−1^, surpassing most previously reported sponge- or foam-based piezoresistive sensors (Fig. 5f and Table S4). The high sensitivity stems from the highly compressible and elastic lamellar structure of CWS@PPy, which enables significant structural deformation even under small pressures and facilitates the rapid formation of highly interconnected conductive networks to lower its internal resistance [40]. At higher pressures (2–12 kPa), the ΔR/R_0_-pressure curve flattens out due to the densification of the lamellar structure with inadequate space for compression, leading to a decrease in sensitivity to 0.06 kPa^−1^. Impressively, the CWS@PPy sensor can monitor a small pressure of only ~ 27 Pa, with a short response time of ~ 400 ms and a recovery time of ~ 300 ms, highlighting its capability for ultra-low-pressure detection (Fig. S29). The stability of the piezoresistive sensor during the cyclic compression process is crucial for its practical applications. The CWS@PPy sensor can maintain a stable current response with slight fluctuation during 8000 loading–unloading cycles at 40% strain, demonstrating its outstanding working stability (Fig. 5g). Furthermore, the sensor can sustain a consistent current response across different loading rates (2.0, 5.8, and 8.6 mm s^−1^) at a constant strain of 40%, confirming its excellent signal stability and loading frequency independence (Fig. 5h). Such remarkable sensing stability can be ascribed to the excellent compressive fatigue resistance of CWS@PPy, which enables long-term repeated compression without damaging its internal structure.

The excellent mechanical flexibility and high piezoresistive sensitivity of CWS@PPy render it an ideal candidate for use as a wearable sensor for human motion detection. As a proof of concept, we evaluated the detection capability of the CWS@PPy sensor for several typical human motions by simply affixing it to human skin. When attached to the finger, the sensor can detect finger bending in real time, producing clear and repeatable current signals corresponding to different bending angles (Fig. 5i). Besides the large motion, the sensor can also monitor subtle physiological activities when affixed to the throat, such as swallowing movements (Fig. 5j), and can distinguish specific vocalizations such as “wood sponge” and “P-P-y” with excellent reproducibility (Fig. 5k). These results highlight the potential of CWS@PPy as a versatile and high-performance pressure sensor for real-time monitoring of human motions.

### Thermal Insulation Performance of CWS@PPy

**Fig. 6:**
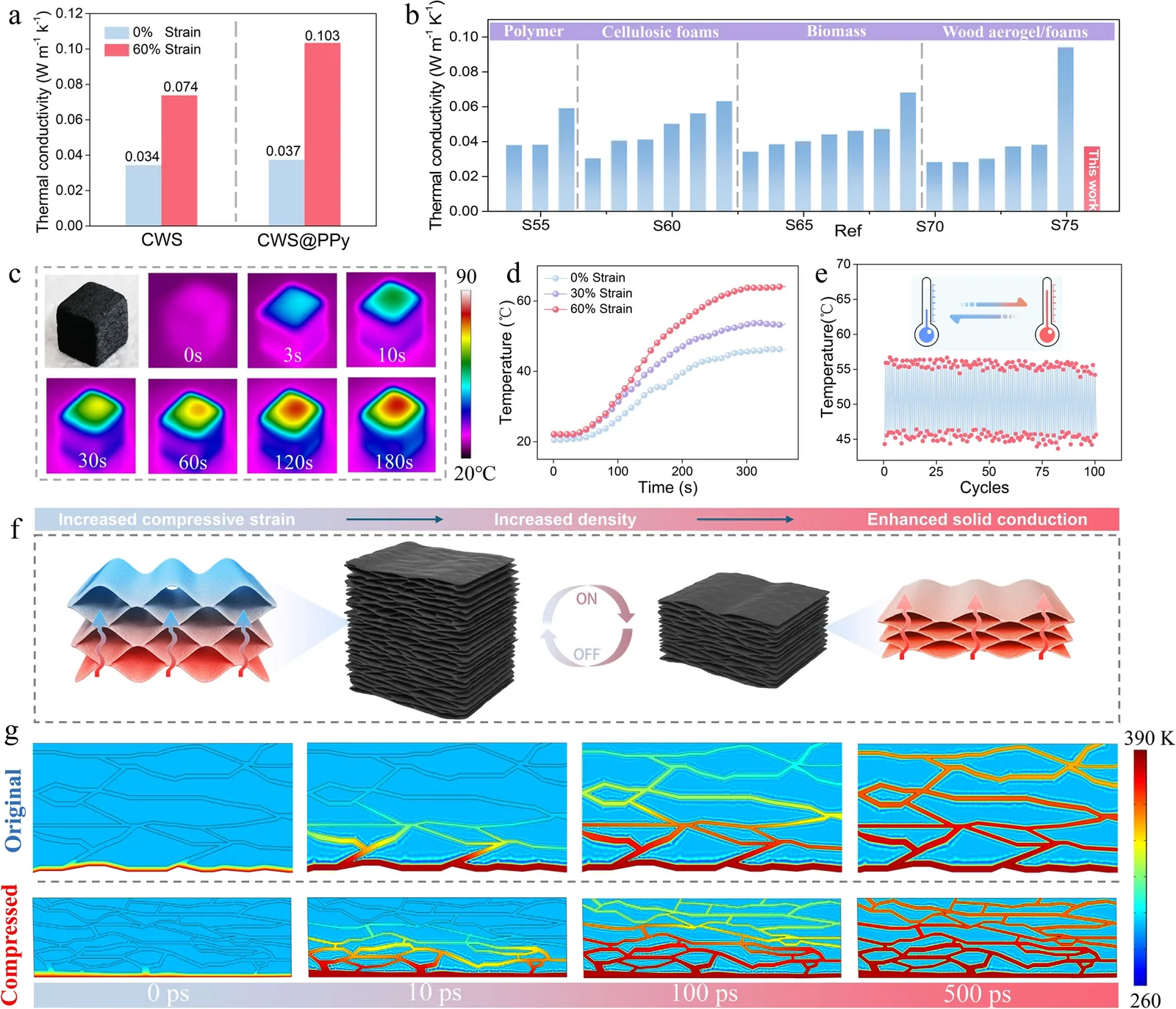
Thermal insulation performance of CWS@PPy. **a** Thermal conductivity of the pristine CWS and CWS@PPy in compressed (60% strain) and uncompressed states. **b** Comparison of thermal conductivity of CWS@PPy with various reported aerogels and foams. **c** Surface temperature of CWS@PPy versus time under xenon light irradiation (1 sun). **d** Time-dependent temperature profile of the top surface of CWS@PPy at varying compressive strains after placing on a 110 °C heating stage. **e** Cyclic stability of the CWS@PPy thermal switch. **f** Mechanism of compression-enhanced heat conduction in CWS@PPy. **g** FEM simulation of heat transfer in CWS@PPy before and after compression

The porous lamellar structure of CWS@PPy makes it highly promising for applications in thermal insulation. As shown in Fig. 6a, the lamellar CWS@PPy exhibited a low thermal conductivity of 0.037 W m^−1^ K^−1^ in the layer-stacking direction (perpendicular to the lamella direction), due to its low density (~ 60 mg cm^−3^) and high porosity. This thermal conductivity value is close to that of the pristine CWS (0.034 W m^−1^ K^−1^), suggesting the negligible impact of the PPy coating and highlighting the critical role of the porous lamellar structure in thermal insulation. Notably, the inherent anisotropic lamellar structure of CWS@PPy imparts a significant anisotropy to its thermal conductivity, which is 2.3 times greater along the fiber direction than in the layer-stacking direction (Fig. S30). The lamellar structure of CWS@PPy facilitates effective heat dissipation along the lamella direction, while simultaneously reducing heat transfer across the aligned channels, thereby resulting in enhanced insulation performance in the layer-stacking direction [48]. The low thermal conductivity of CWS@PPy is comparable to those of previously reported wood- and cellulose-based foams and aerogels (Fig. 6b and Table S5). To visualize the thermal insulation performance of CWS@PPy in the layer-stacking direction, an infrared thermal camera was used to track the surface temperature changes over time under xenon light irradiation (1 sun). The top surface of the sample was rapidly heated to ~ 72 °C within 2 min from its initial ~ 23 °C due to the excellent photothermal conversion capability of PPy, whereas the underlying structure maintained a relatively low-temperature state, indicating minimal heat dissipation in the layer-stacking direction and confirming its excellent thermal insulation performance (Fig. 6c).

Leveraging its high compressive elasticity, the thermal conductivity of CWS@PPy can be dynamically tuned by simply varying the compressive strain, offering a promising strategy for smart thermal management. As the compressive strain increased from 0 to 60%, the thermal conductivity increased from 0.037 to 0.103 W m^−1^ K^−1^, suggesting its compression-enhanced heat conduction (Fig. 6a). A similar trend is observed in the pristine CWS, although the increase is less pronounced, highlighting the amplifying role of the conductive PPy coating. To further validate the tunable heat transfer, we recorded the upper surface temperature changes in real time using a homemade testing system (Fig. S31). The sample was placed on a 110 °C hot plate and then compressed to 30% and 60% strains during measurement (Fig. 6d). The surface equilibrium temperature increased from ~ 45 °C (uncompressed) to ~ 54 °C (30% strain) and reached ~ 65 °C (60% strain), confirming the compression-enhanced heat transfer. To demonstrate the reversible thermal conductivity switching, we defined the uncompressed CWS@PPy as heat “insulation” mode and the 30% strain as heat “dissipation” mode based on the relative equilibrium temperature. As shown in Fig. 6e, the reversible modulation of surface temperature can be achieved by repeated compression and decompression. Notably, the peak and valley temperatures maintained steady at ~ 56 and ~ 45 °C over 100 cycles, demonstrating its excellent long-term stability.

We propose that this compression-tunable thermal behavior of CWS@PPy arises from the dynamically tunable heat transfer pathways (Fig. 6f) [49, 50]. For the uncompressed CWS@PPy, the large interlayer gaps and the abundant micro/nanopores within the lamellas promotes the interfacial phonon scattering and reduce the solid conduction, thus contributing to its low thermal conductivity. Upon compression, air is largely expelled from the porous scaffold, resulting in a densified scaffold with lowered porosity and enhanced density (Fig. S32). Such densified scaffold can prominently reduce the interfacial thermal resistance and enhance the solid conduction by generating more interfacial contacts between the adjacent lamellas, thus facilitating efficient heat transfer and enhancing the thermal conductivity in the layer-stacking direction [51, 52]. Finite element modeling (FEM) further supports this compression-enhanced heat transfer effect (Fig. 6g and Video S3). The structural models derived from the SEM images of CWS@PPy were subjected to a simulated heat source at 110 °C. The simulations revealed greatly enhanced heat transfer and higher upper surface temperature in the compressed state compared to the uncompressed state over the same time period, in line with the experimental results. These findings demonstrate that CWS@PPy can function as a mechanically tunable thermal switch, enabling dynamic modulation between the thermal insulation and heat dissipation modes, with strong potential for smart thermal management in intelligent electronics and energy systems [51, 53–55].

### Multifunctional Integration for Practical Application of CWS@PPy

Our CWS@PPy demonstrates compelling functionalities in switchable EMI shielding, pressure sensing, and smart thermal management. The seamless integration of these features into a reliable smart material is imperative for practical applications. Here, we take tunable EMI shielding and smart thermal management as two representative functions to illustrate the advantages of our material in real-world applications (Fig. S33). As a typical EMI shielding material, CWS@PPy not only exhibit excellent shielding performance but also provides thermal insulation due to its porous structure. However, during the shielding process, a portion of EM waves can still penetrate the material and be converted into thermal energy. Notably, most previously reported 3D porous EMI shielding materials possess constant thermal conductivity due to their fixed pore structures, which may lead to the accumulation of internally generated heat and potential damage to the protected electronic components. In contrast, our CWS@PPy features dynamically tunable thermal conductivity, enabled by compression-mediated regulation of its internal thermal conduction pathways. When heat builds up within the material, mild compression can enhance its thermal conductivity to facilitate rapid heat dissipation, thereby effectively mitigating the risk of local overheating. This capability underscores the unique advantage of CWS@PPy in real-world electromagnetic protection systems.

## Conclusion

In summary, we developed a highly elastic, fatigue-resistant, and electrically conductive lamellar wood sponge based on a sustainable “top-down” cell wall reconfiguration strategy. By transforming the intrinsic wood cellular structure into an arched-shaped lamellar structure at the microscale and introducing covalent cross-linking in the lamella at the nanoscale, we obtained a structurally robust and highly elastic wood sponge. The subsequent in situ deposition of a continuous, uniform, and stable PPy nanocoating (~ 200 nm thick) on the lamellar scaffold of CWS endowed it with favorable electrical conductivity, which can be dynamically regulated by varying the compressive strain. The resultant CWS@PPy exhibited reversible compressibility and high fatigue resistance (∼3.5% plastic deformation after 10,000 cycles at 40% strain). The strain-sensitive electrical conductivity enabled tunable EMI shielding performance of CWS@PPy with a stable and reversible switch between a high shielding state (~ 75 dB) at the uncompressed state and a low shielding state (~ 34 dB) at 60% compressive strain for 6000 cycles. The CWS@PPy at its uncompressed state exhibited a high specific EMI SE of 1286 dB cm^−3^ g^−1^, surpassing most previously reported porous shielding materials. The high specific EMI SE, broad tunable range, and exceptional switching stability of CWS@PPy make it a highly competitive candidate for tunable EMI shielding applications. Leveraging its stress-sensitive electrical conductivity, CWS@PPy can also function as a highly responsive piezoresistive sensor with excellent pressure sensitivity (0.72 kPa^−1^), low detection threshold (~ 27 Pa), and long-term working stability (> 8000 cycles). Moreover, the porous lamellar structure endowed the material with a low through-plane thermal conductivity of 0.037 W m^−1^ K^−1^ , which is compression-tunable for smart thermal management. Overall, this work offers an innovative top-down strategy for fabricating anisotropic lamellar CEMs with high compressive elasticity, excellent fatigue resistance, and tunable conductivity for multifunctional applications, including switchable EMI shielding, pressure sensing, and smart thermal management.

## Supplementary Information

Below is the link to the electronic supplementary material.

Supplementary file1 (DOCX 30878 KB)

Supplementary file2 (MP4 1389 KB)

Supplementary file3 (MP4 1940 KB)

Supplementary file4 (MP4 1079 KB)
