# Supplementary material for: Highly Elastic and Conductive Lamellar Wood Sponge via Cell Wall Reconfiguration Toward Smart Multifunctional Applications
Source: Nanomicro Lett. 2026 Jan 5;18:171. doi: 10.1007/s40820-025-02016-4 (PMC12765785; doi:10.1007/s40820-025-02016-4)
Supplement: Supplementary file 1 — Supplementary file1 (DOCX 30878 KB) [file 40820_2025_2016_MOESM1_ESM.docx]

Supporting Information for

**Highly Elastic and Conductive Lamellar Wood Sponge** **via** **Cell Wall Reconfiguration Toward Smart Multifunctional Applications**

Xin-jian Dai^1^, Xin Wang^1^, Ji-hang Hu^1^, Pan Jiang^1^*, Xiao-qing Wang^1^*

^1^Research Institute of Wood Industry, Chinese Academy of Forestry, Xiangshan Road, Haidian District, Beijing 100091, P. R. China

*Corresponding authors. E-mail: [jiangpan@caf.ac.cn](mailto:jiangpan@caf.ac.cn) (Pan Jiang); [wangxq@caf.ac.cn](mailto:wangxq@caf.ac.cn) (Xiao-qing Wang)

**Supplementary Figures and Tables**





**Fig. S1** The multiscale cell wall engineering strategy for CWS fabrication


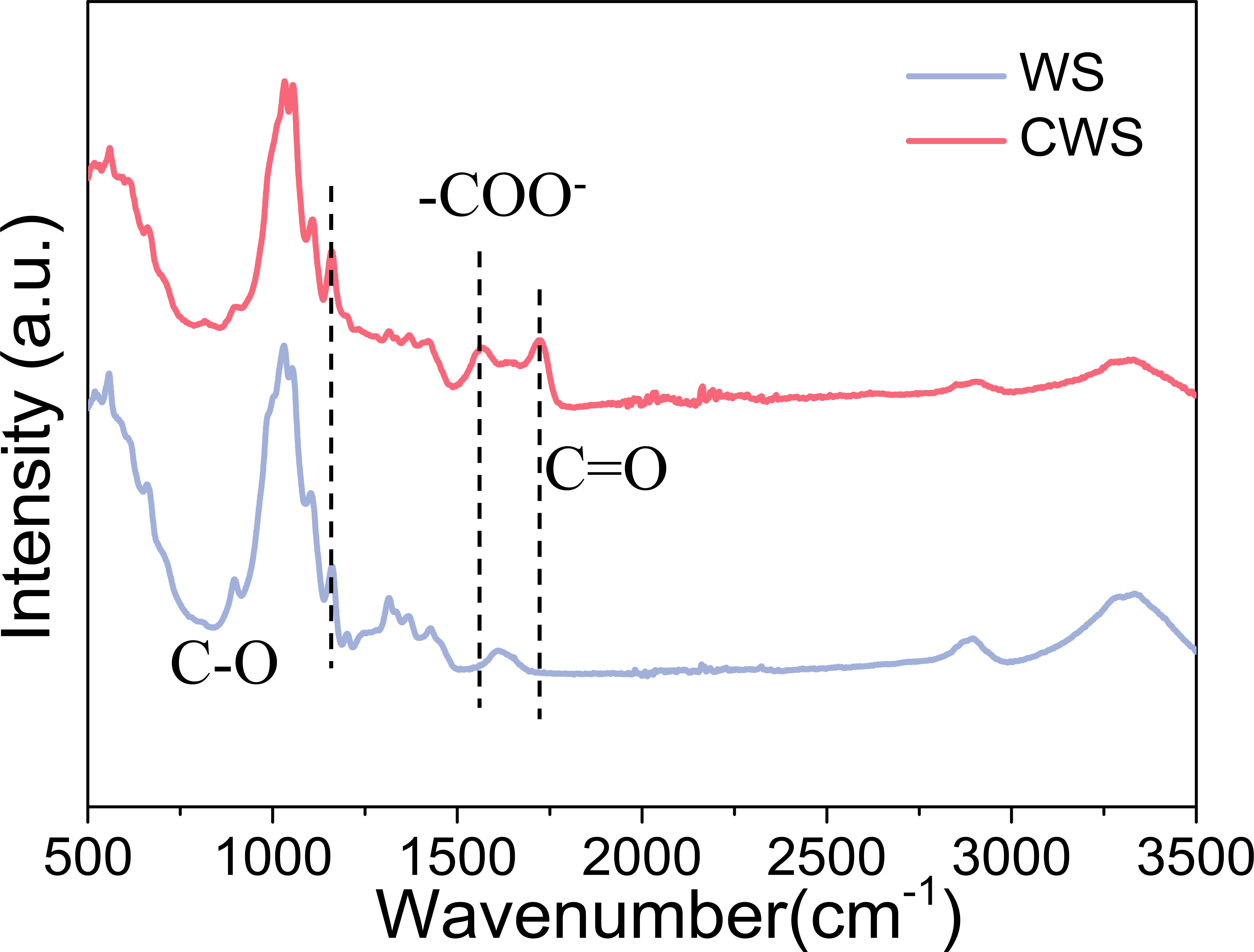


**Fig. S2** FTIR spectra of WS and CWS


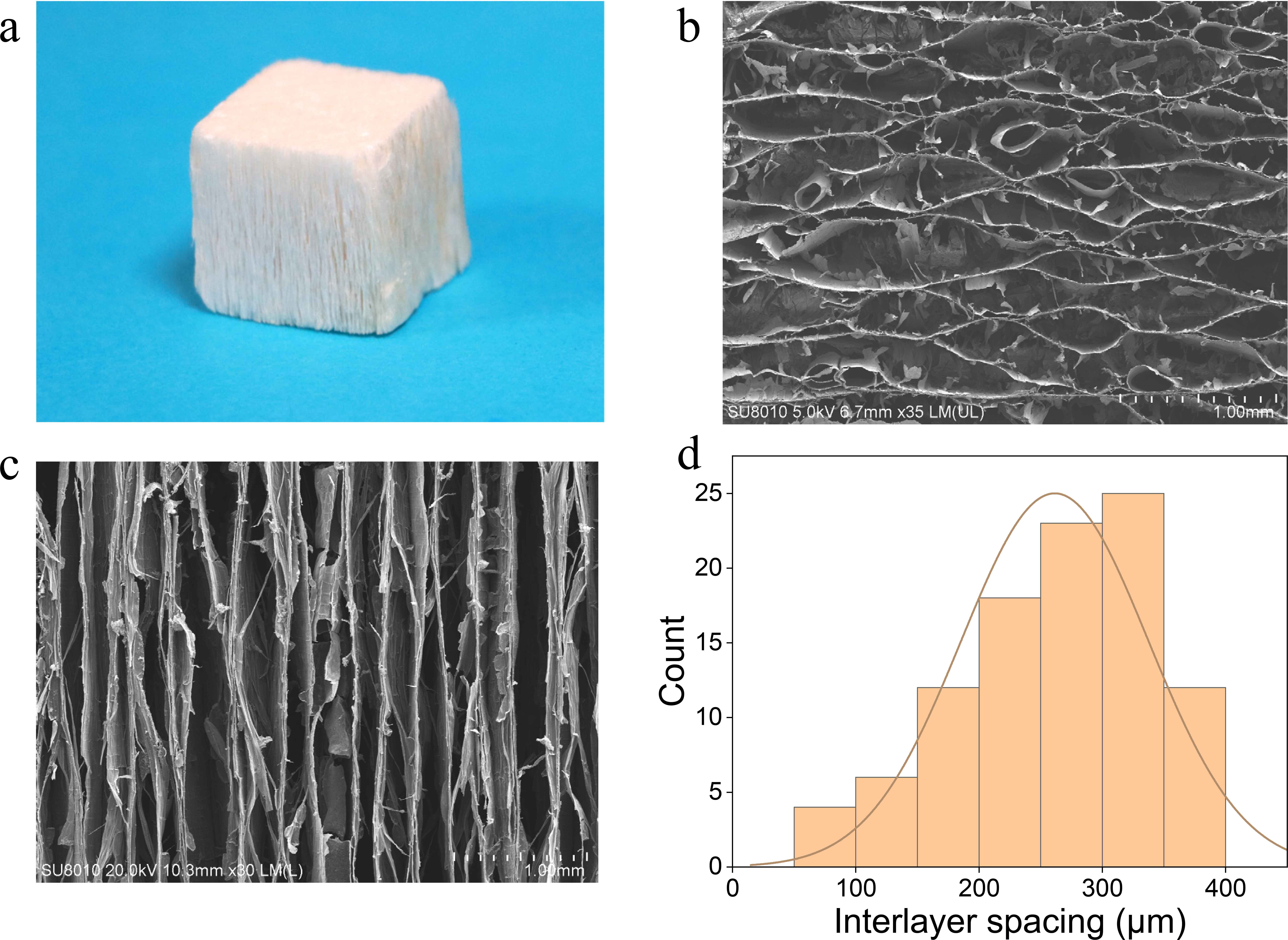


**Fig. S3** Photograph (**a**), cross section (**b**), tangential section (**c**) of CWS and its distribution of interlayer spacing (**d**)


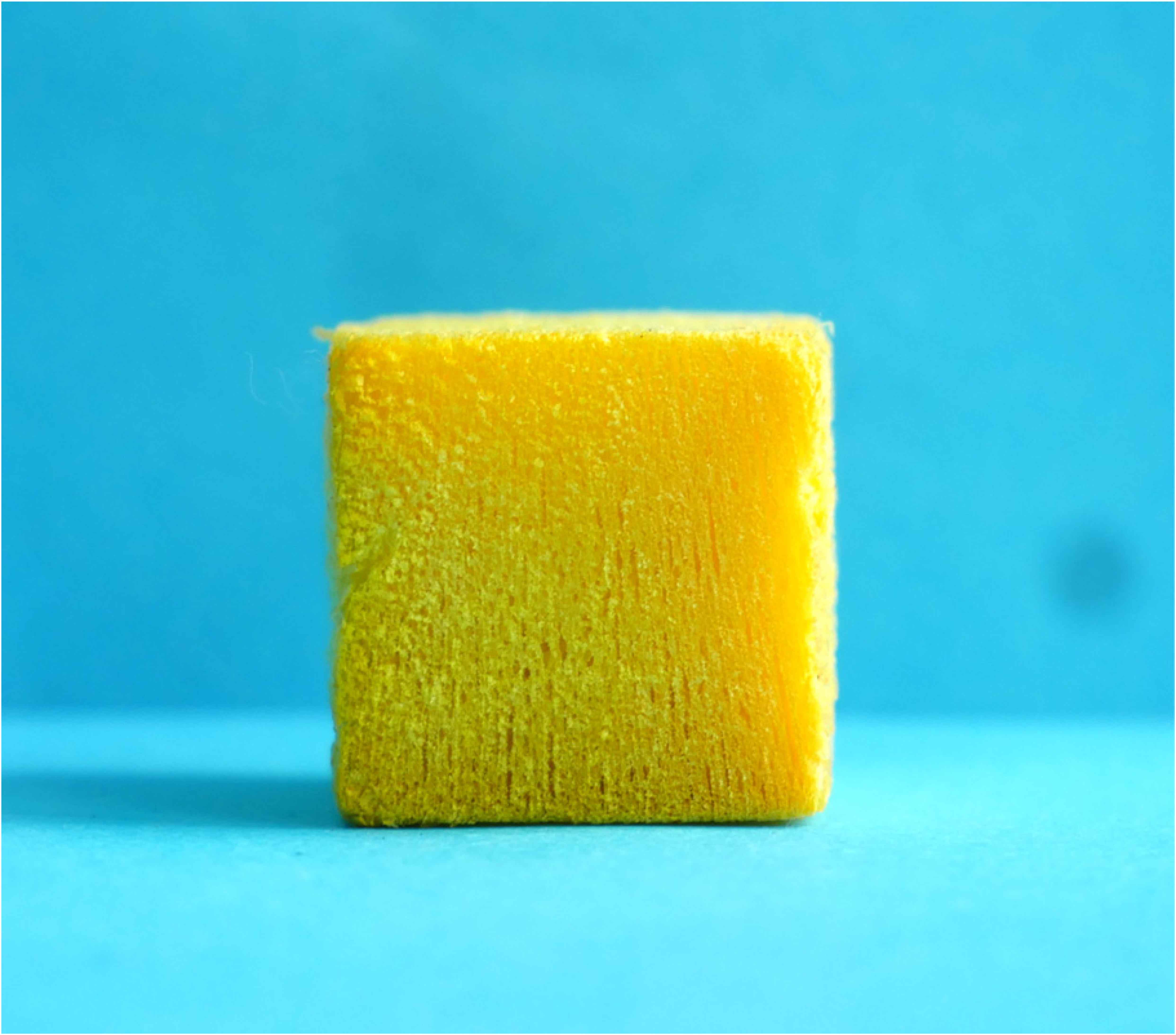


**Fig. S4** Photograph of CWS after chelating with Fe^3+^ ion

**
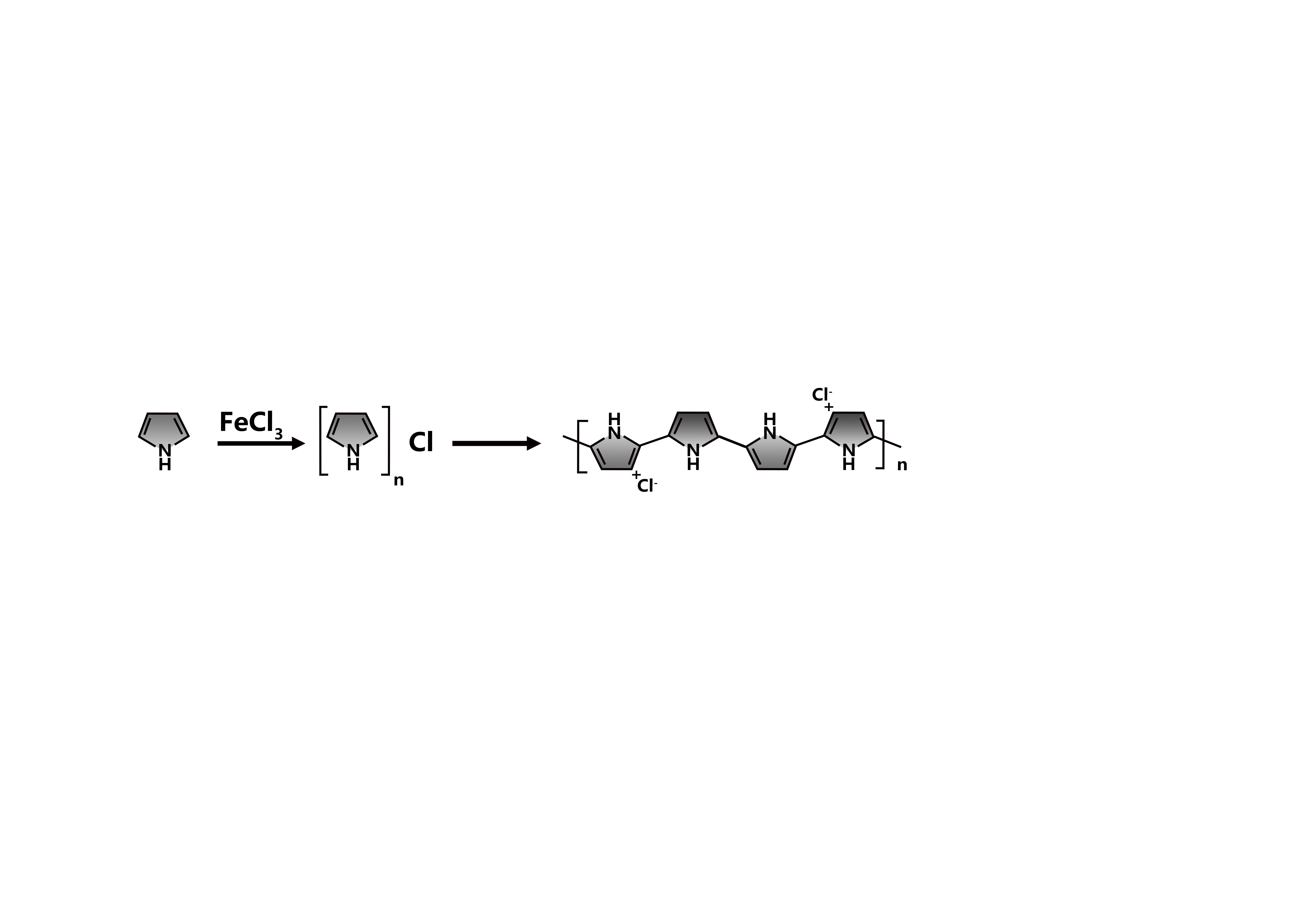
**

**Fig. S5** The reaction mechanism for the oxidative polymerization of Pyrrole monomers


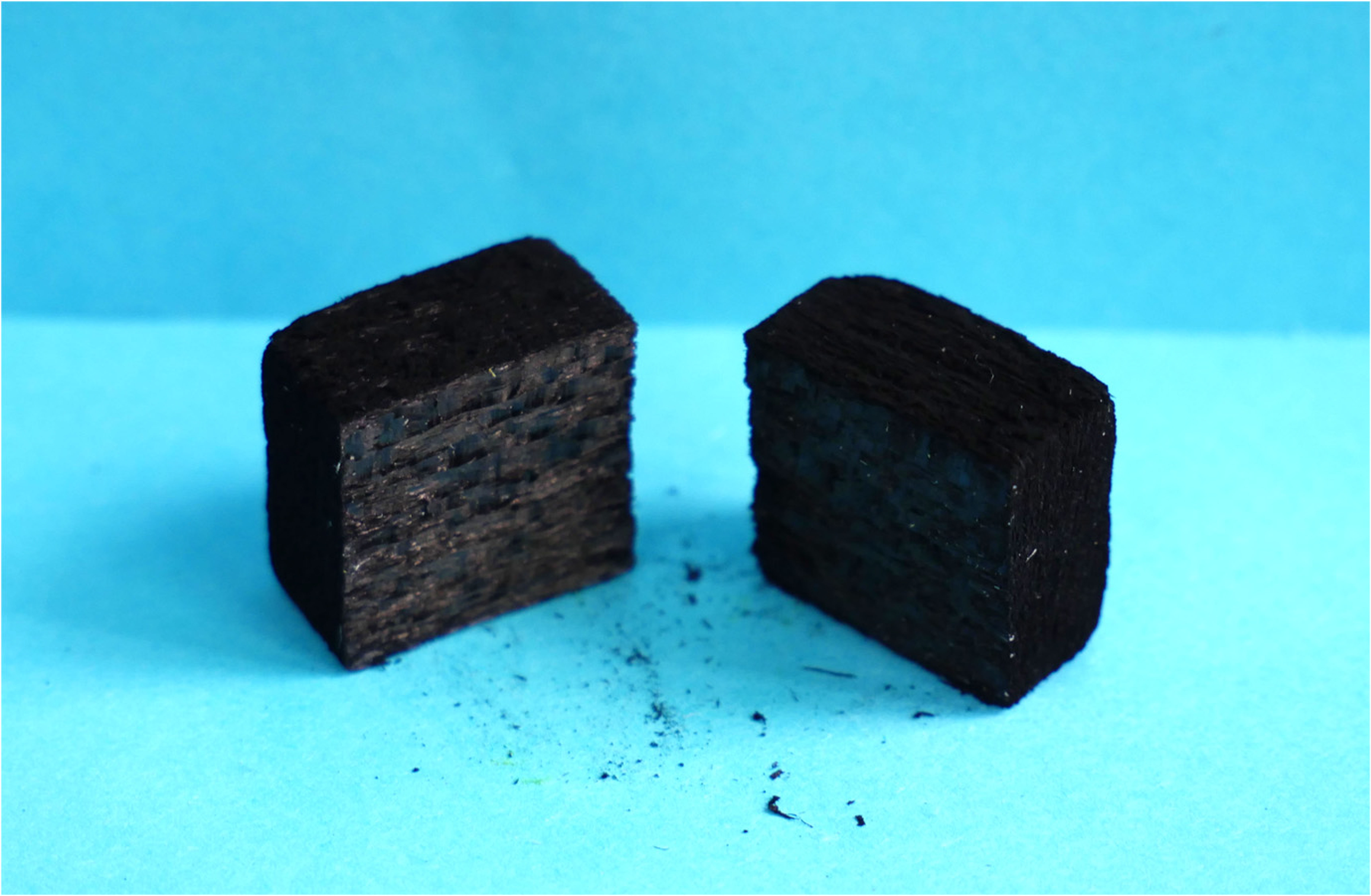


**Fig. S6** Photograph of CWS@PPy after being cut in half


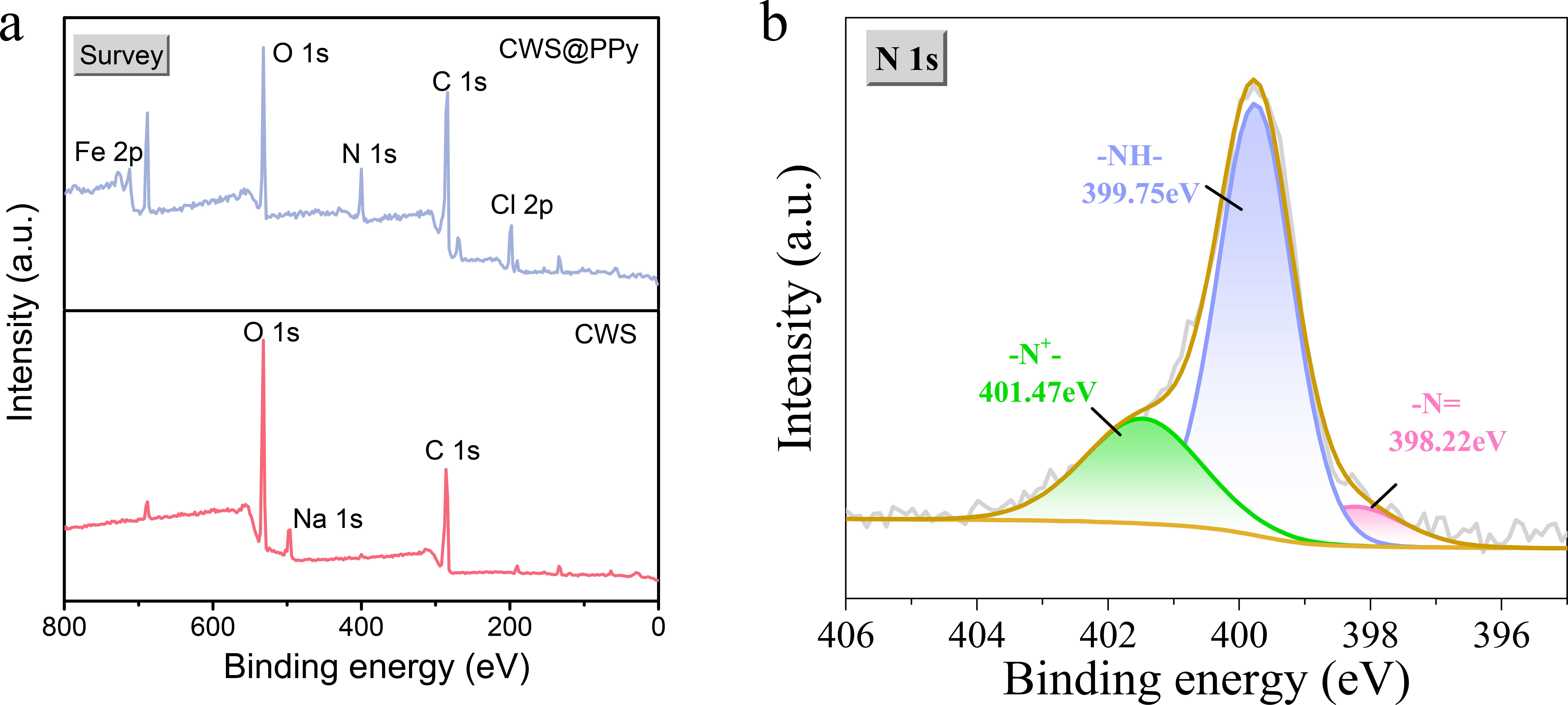


**Fig. S7** XPS survey spectra (**a**) and N 1s spectrum (**b**) of samples


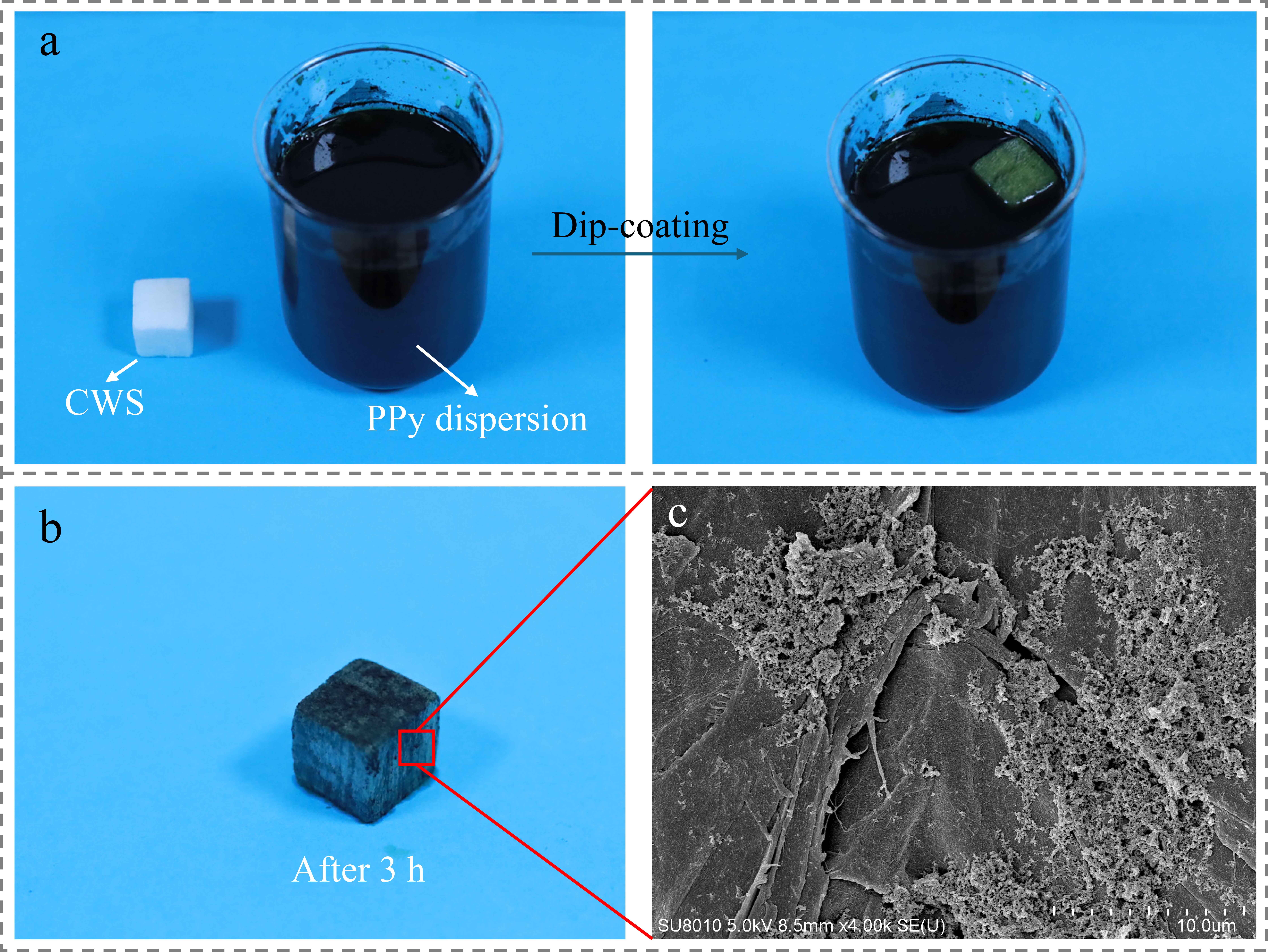


**Fig. S8** Photographs of CWS@PPy prepared via the dip-coating method (**a-b**) and SEM image of the sample (**c**)


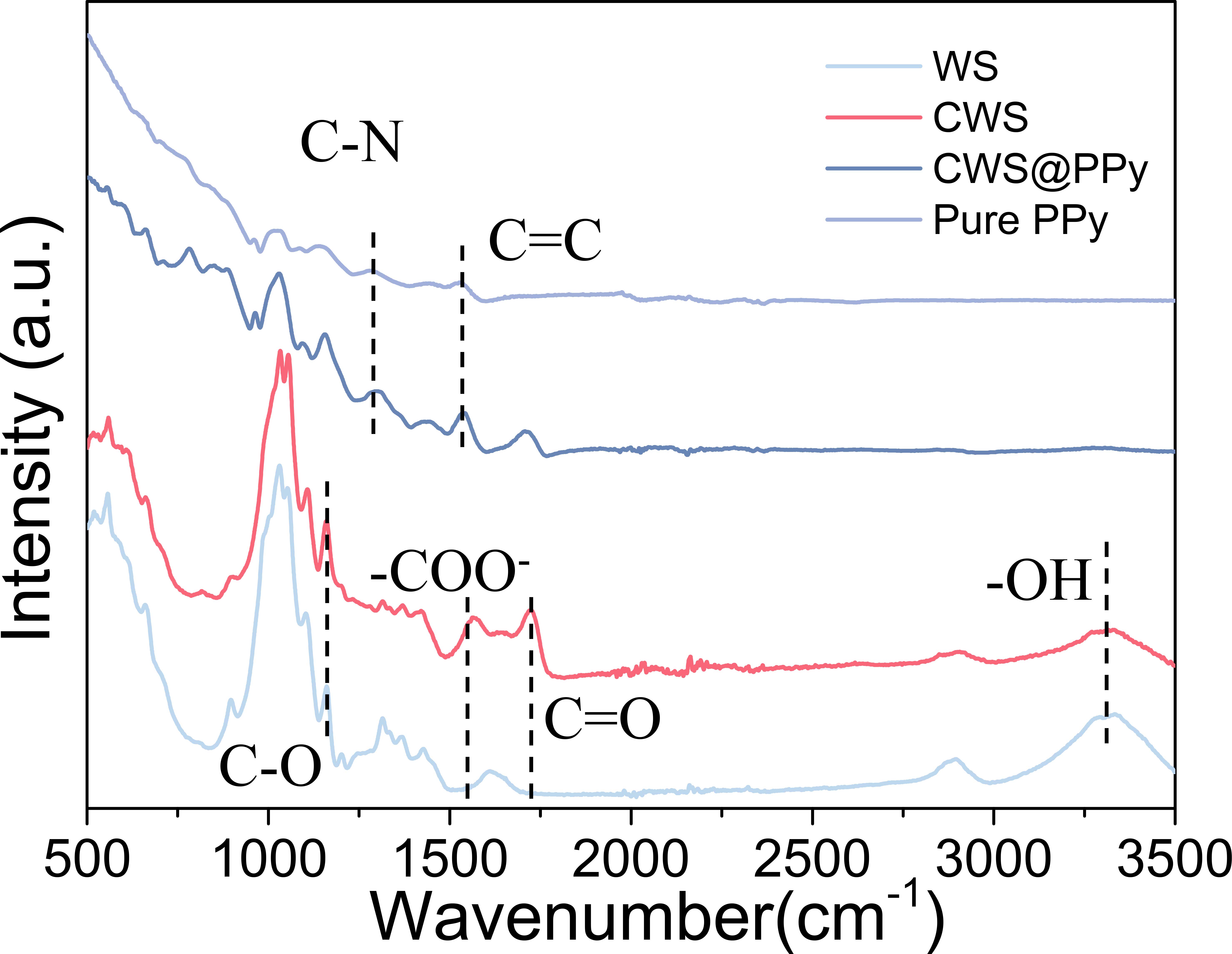


**Fig. S9** FTIR spectra of various samples


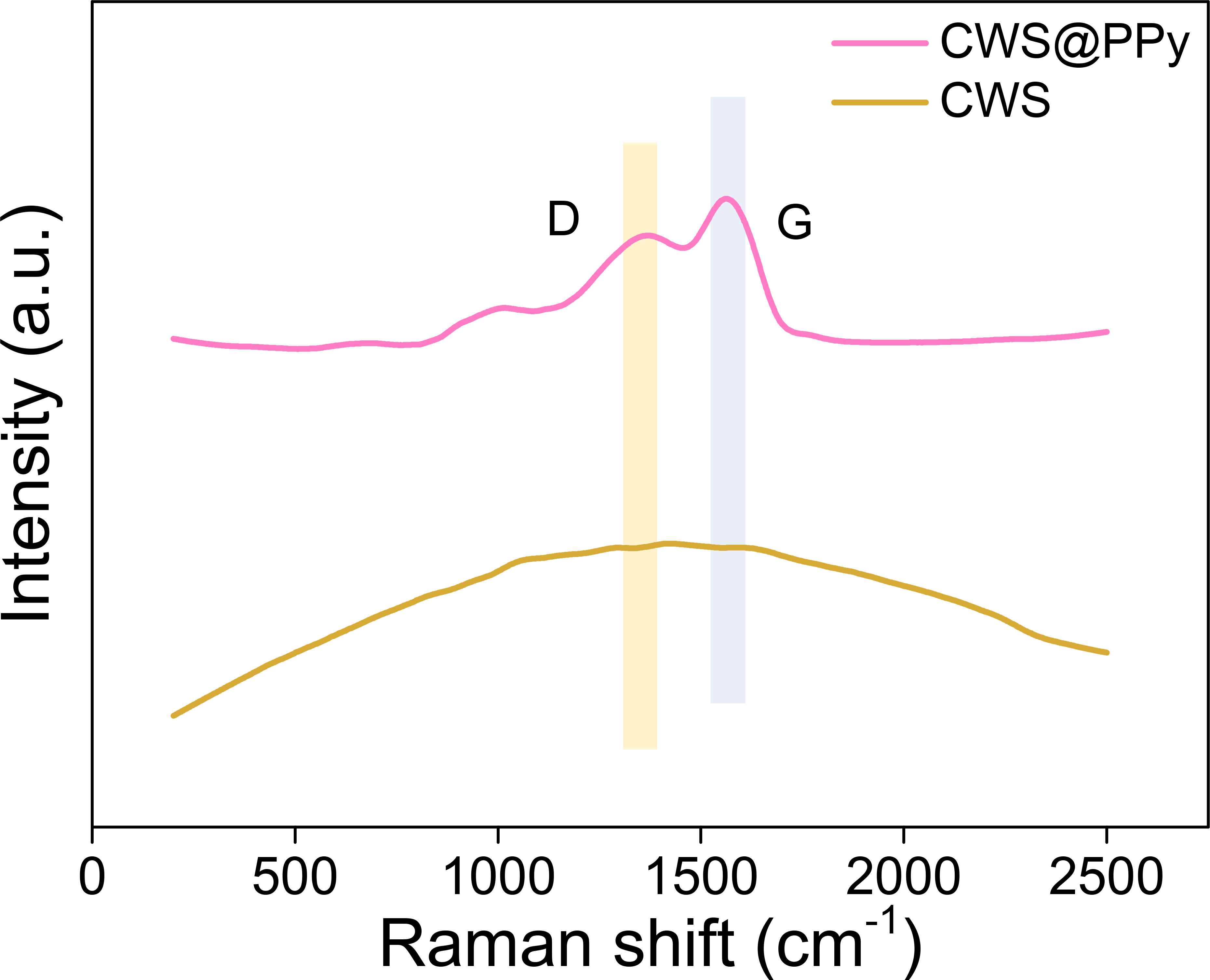


**Fig. S10** Raman spectra of CWS and CWS@PPy


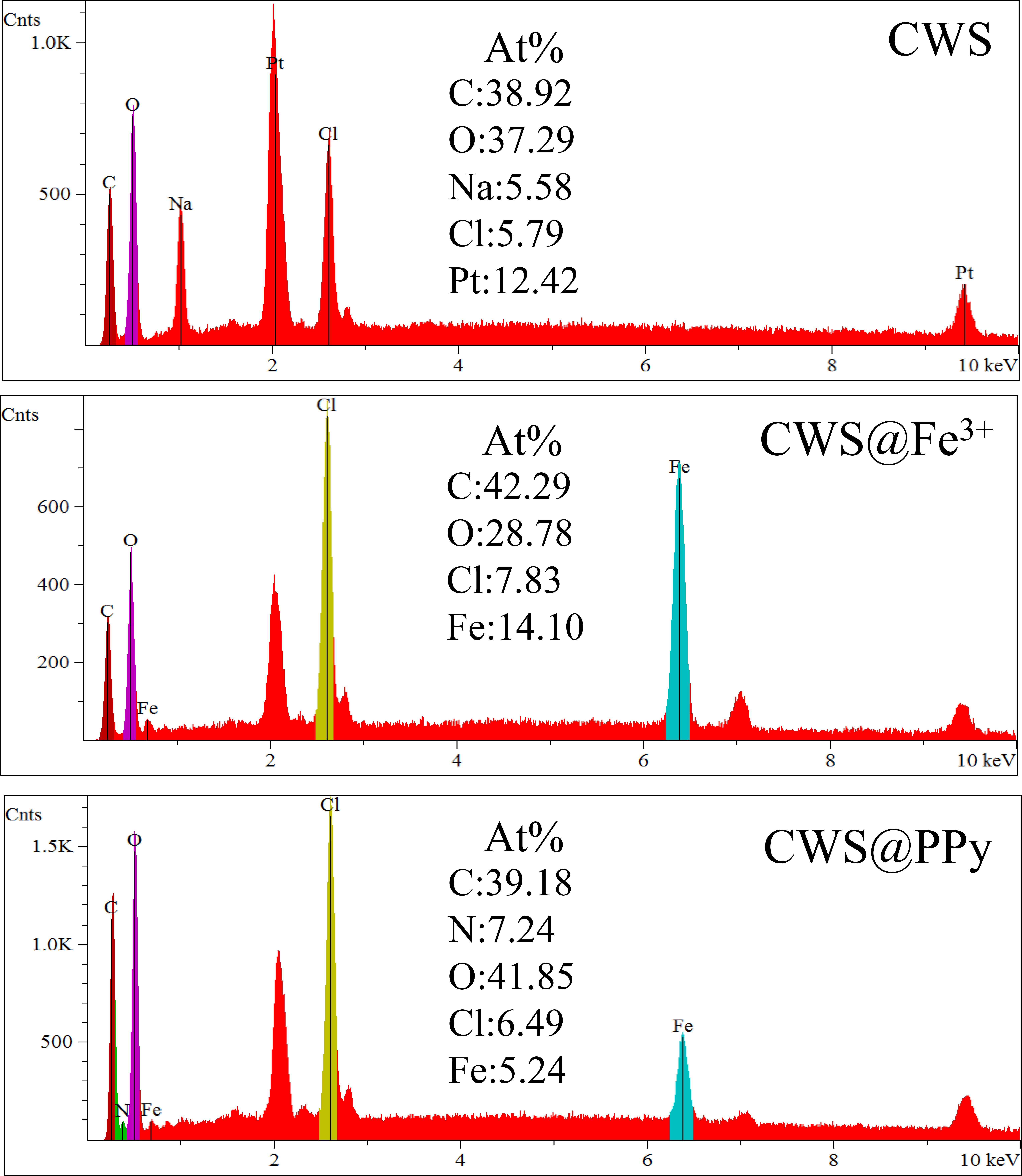


**Fig. S11** The element compositions of CWS, CWS@Fe^3+^, and CWS@PPy


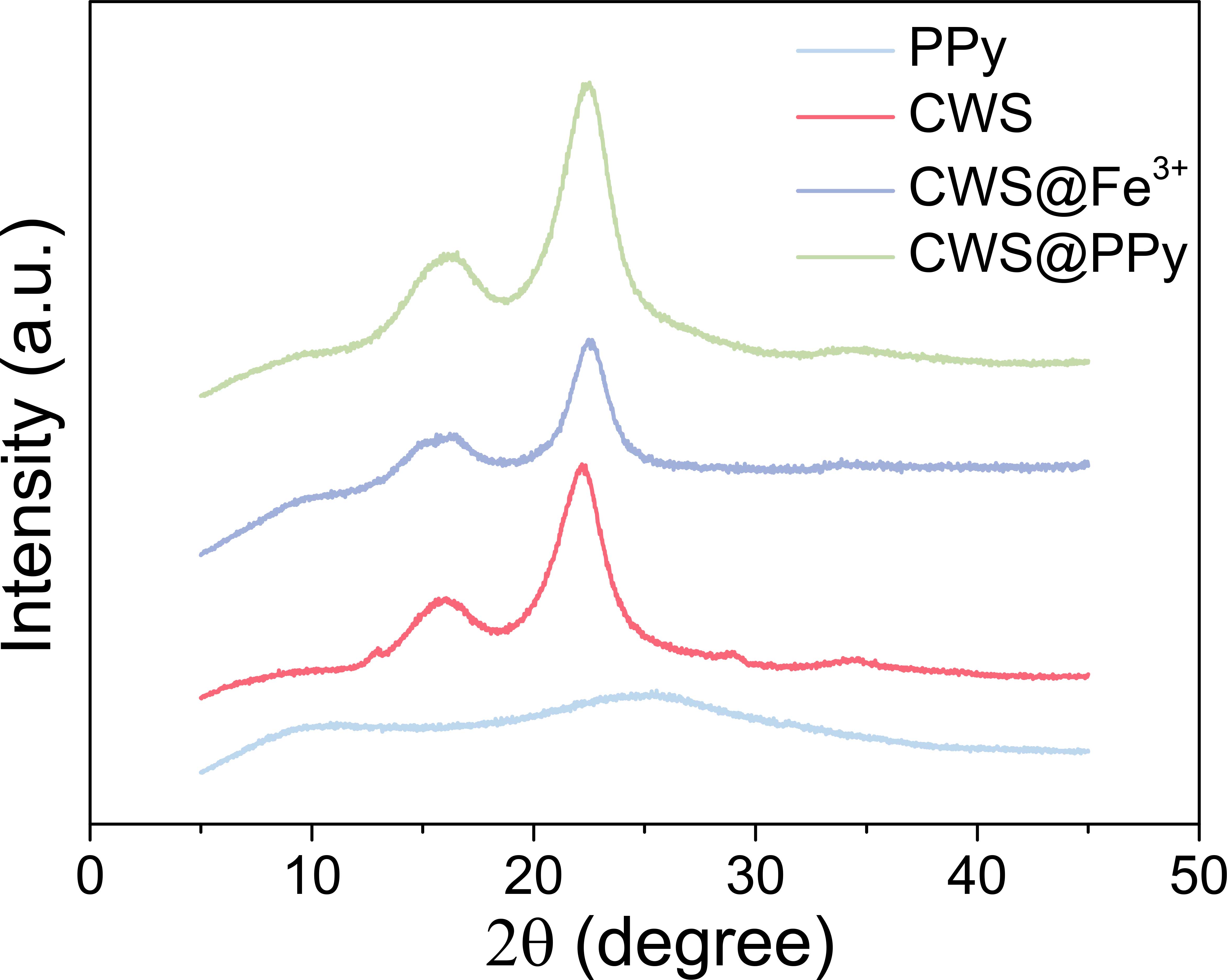


**Fig. S12** The X-ray diffraction patterns of various samples

**
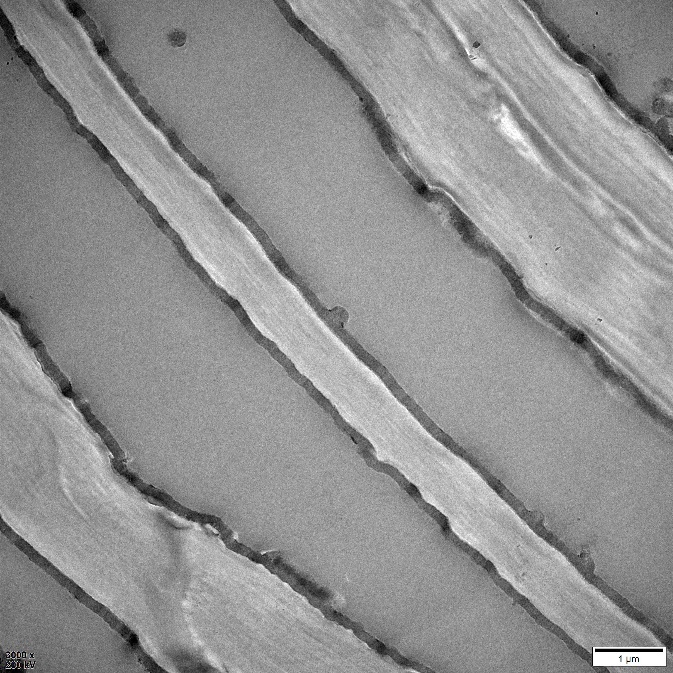
**

**Fig. S13** TEM image showing the continuous PPy nanocoating on the surface of the microfiber bundle


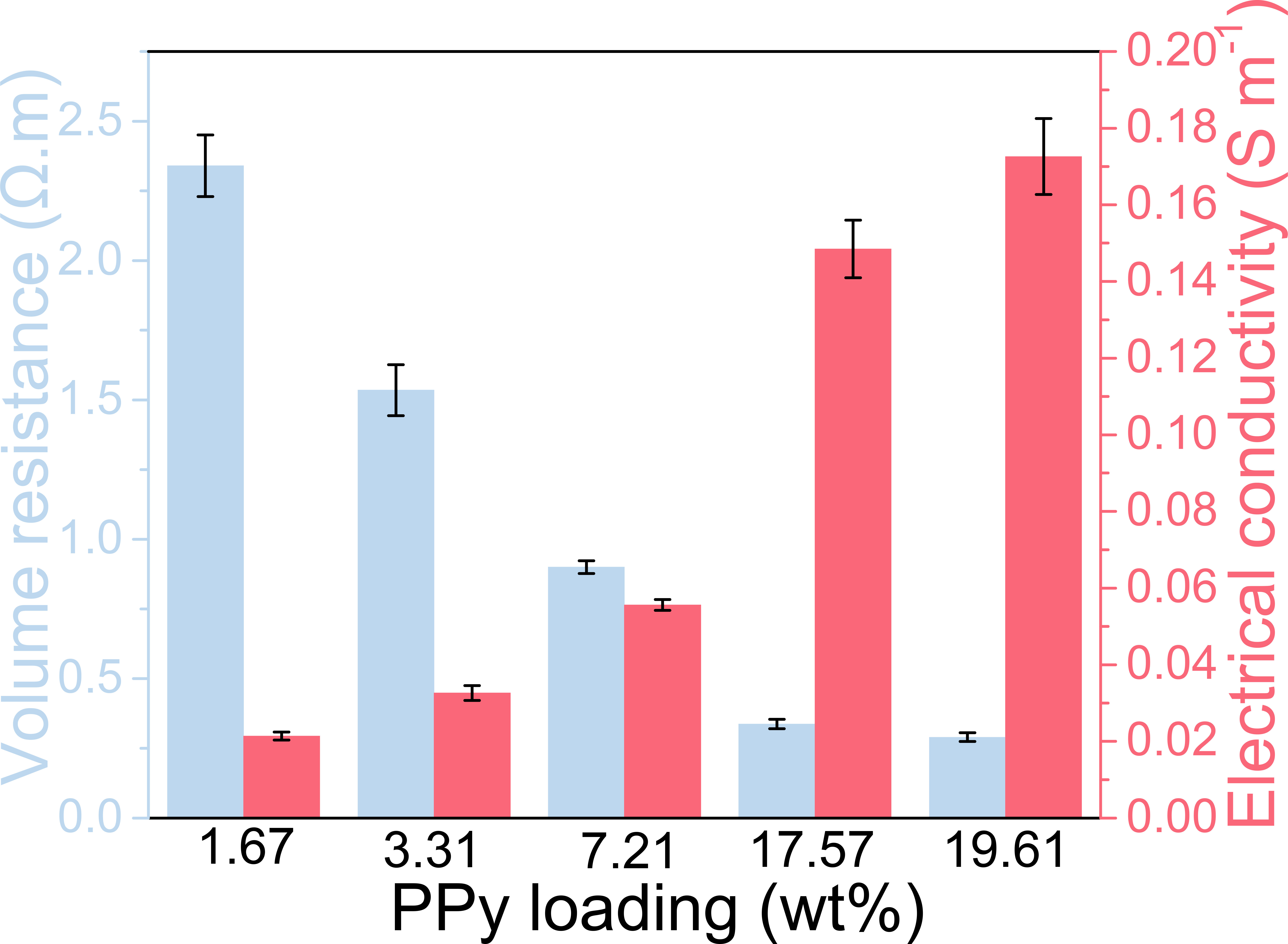


**Fig. S14** The volume resistance and electrical conductivity of CWS@PPy with different PPy loading





**Fig. S15** SEM images of radial section of CWS@PPy with different polymerization durations


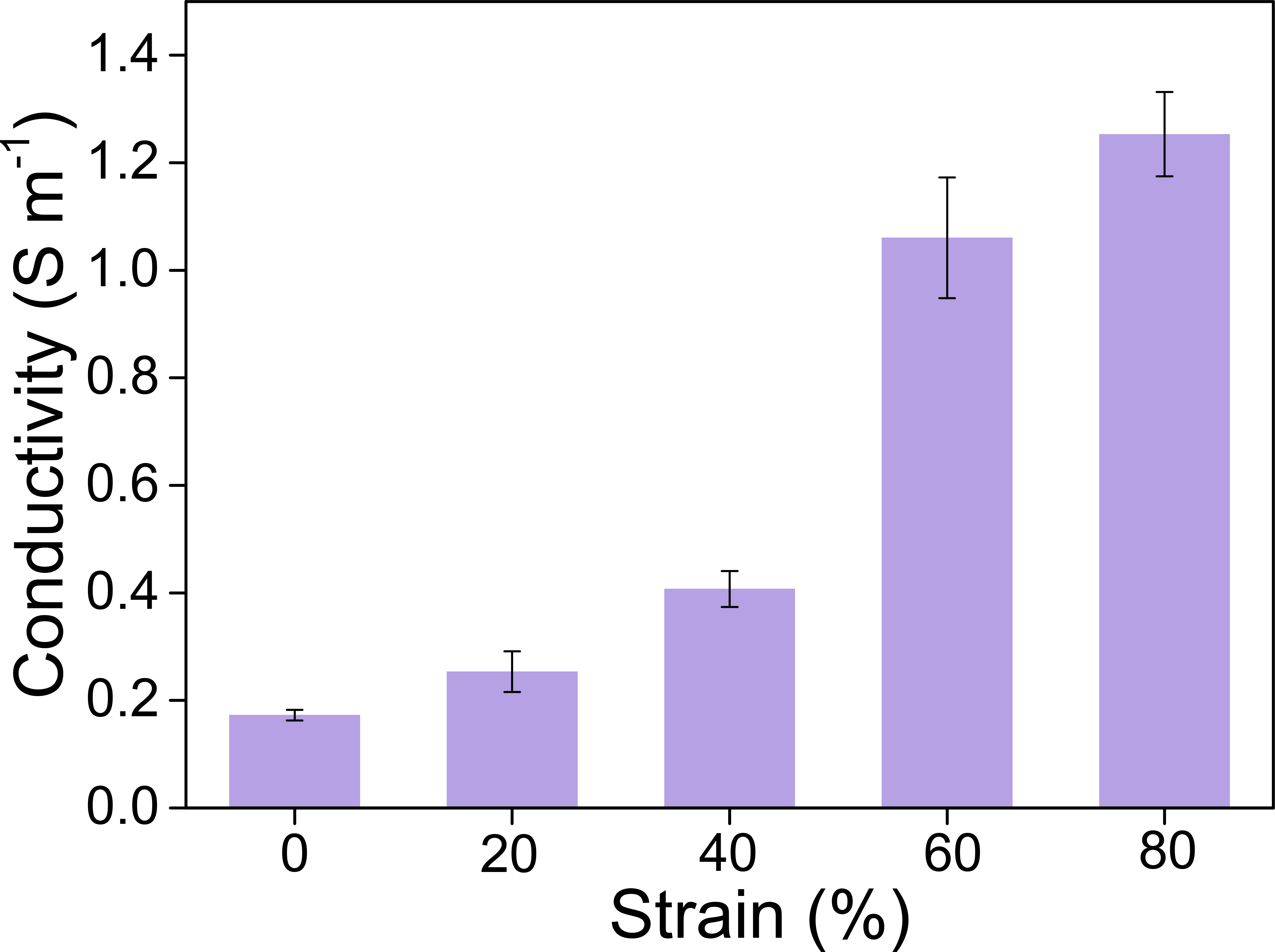


**Fig. S16** Electrical conductivity of CWS@PPy (19.61 wt%) with different strain


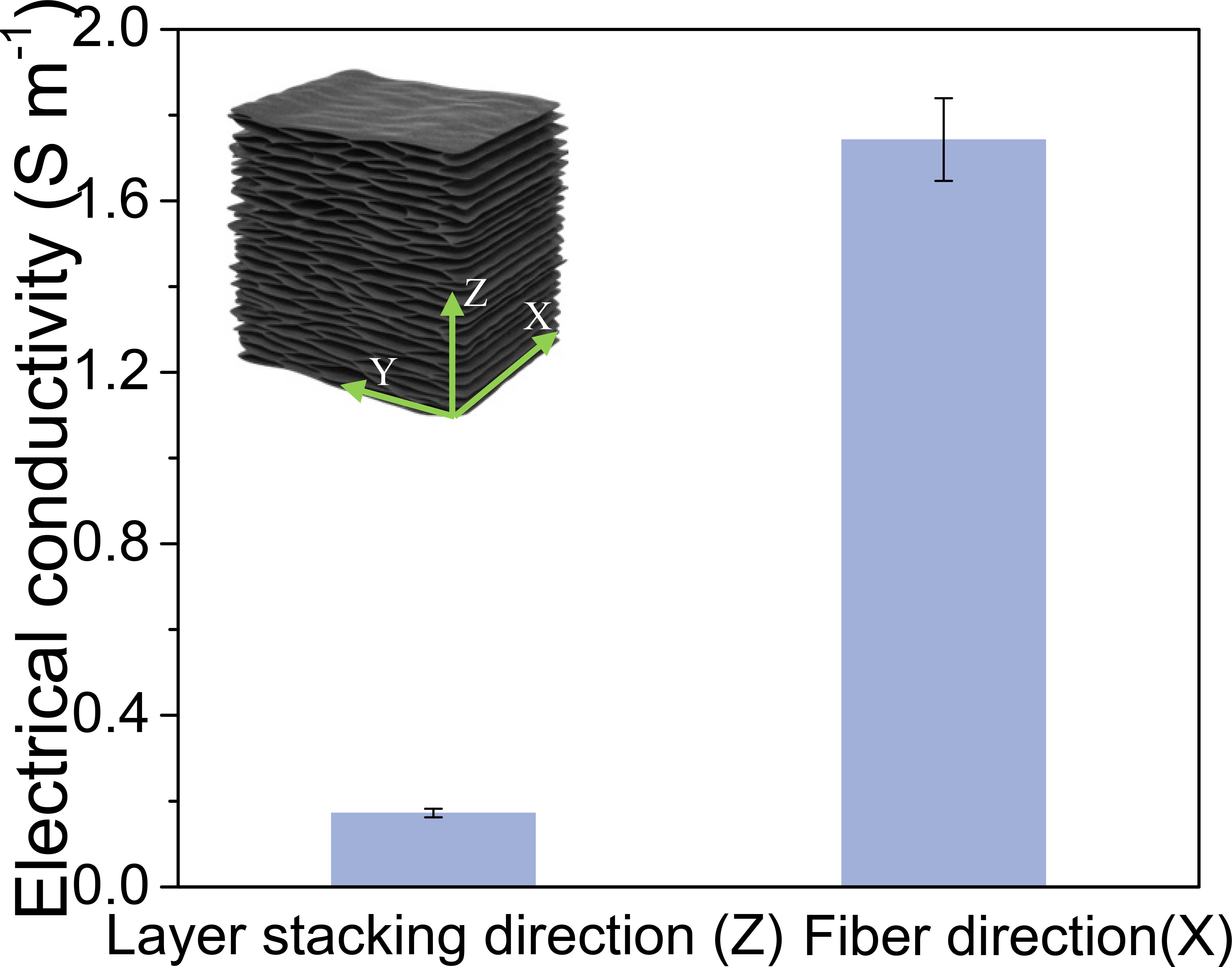


**Fig. S17 A**nisotropic electrical conductivity of CWS@PPy





**Fig. S18** Photographs of aqueous solutions containing CWS@PPy subjected to ultrasonication d) SEM image of the sample after ultrasonic treatment for 6h





**Fig. S19** The acid/alkali tolerance of CWS@PPy


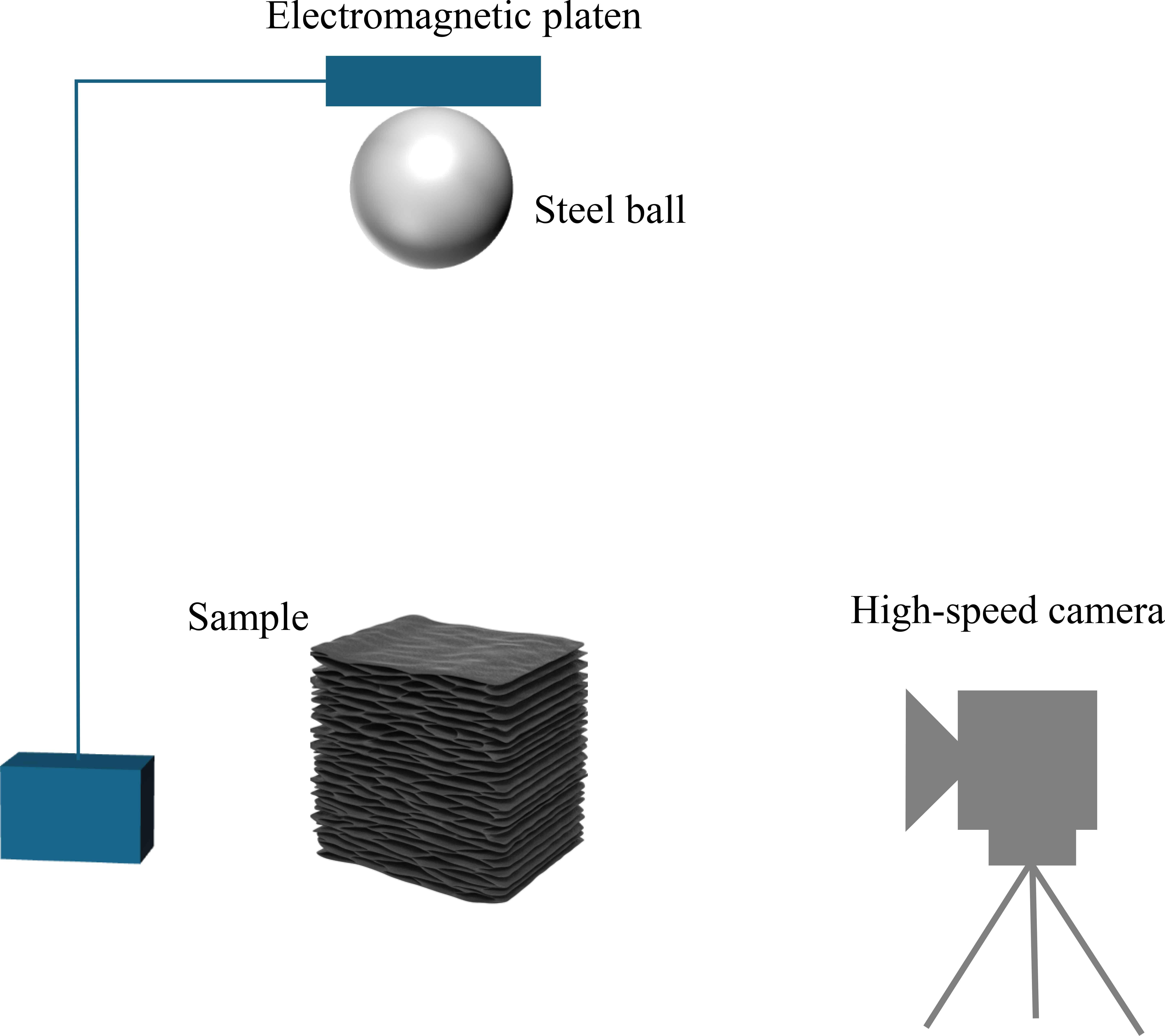


**Fig. S20** Schematic diagram of the dynamic impact testing system for the sample


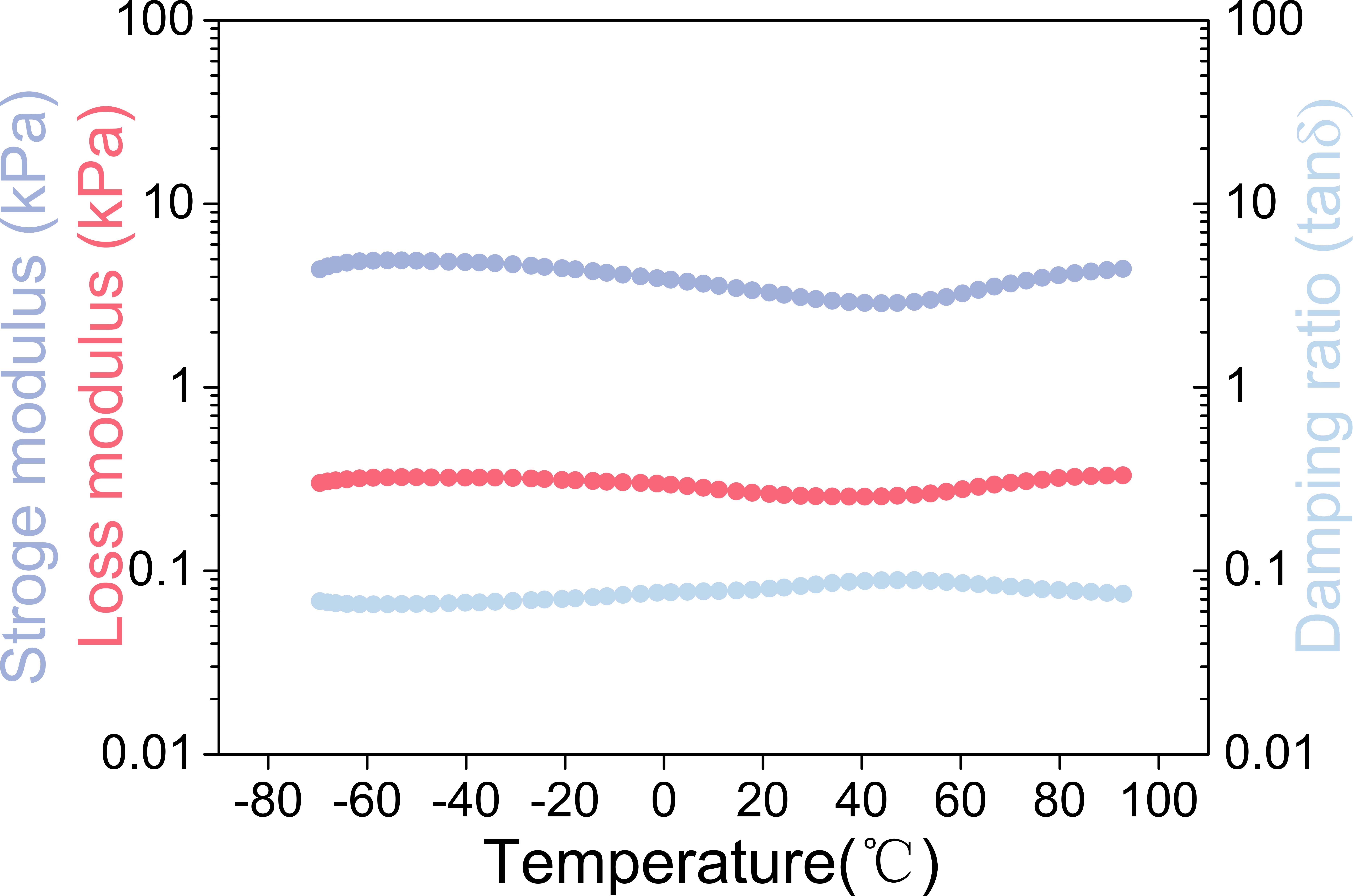


**Fig. S21** Viscoelastic properties (storage modulus, loss modulus, and damping ratio) of CWS as a function of temperature (-70 to 90 °C)


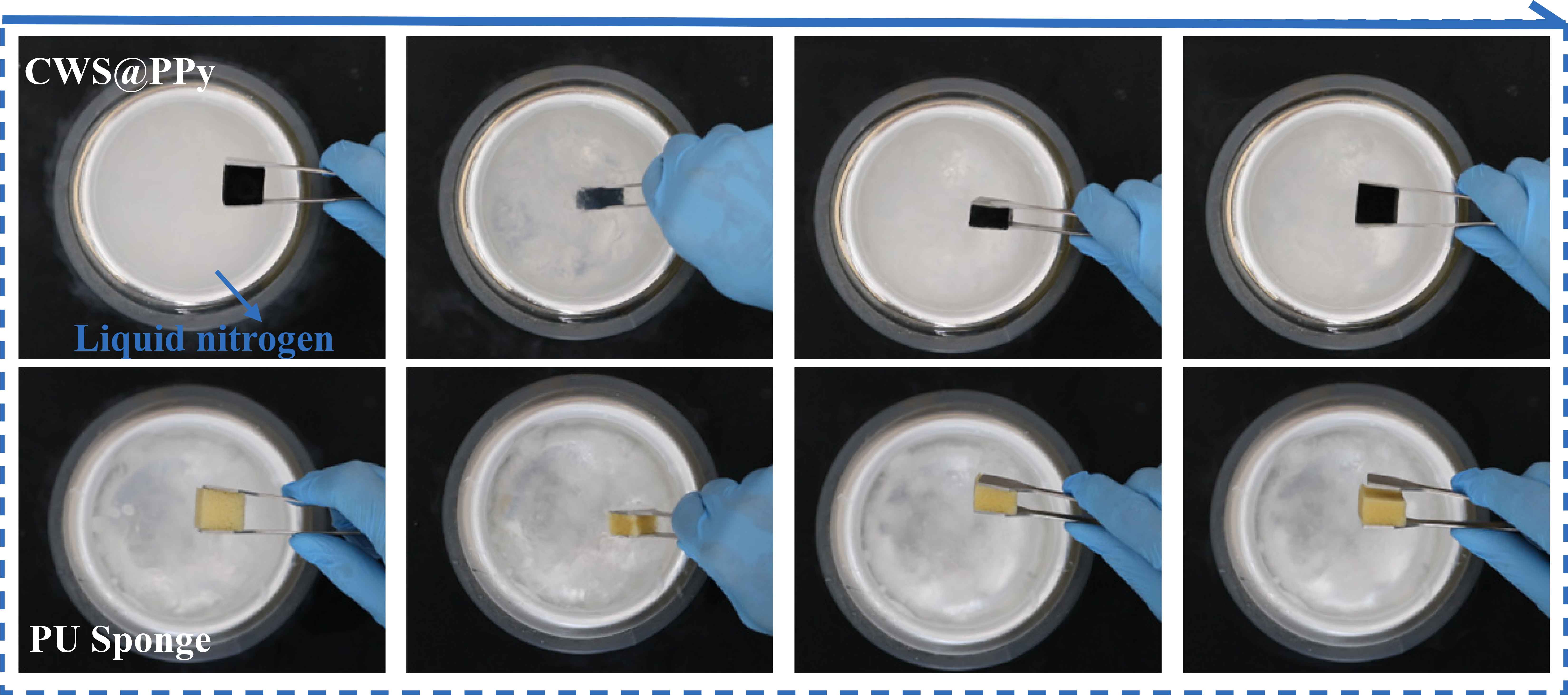


**Fig. S22** Comparison of compressive elasticity of CWS@PPy and PU sponge in liquid nitrogen


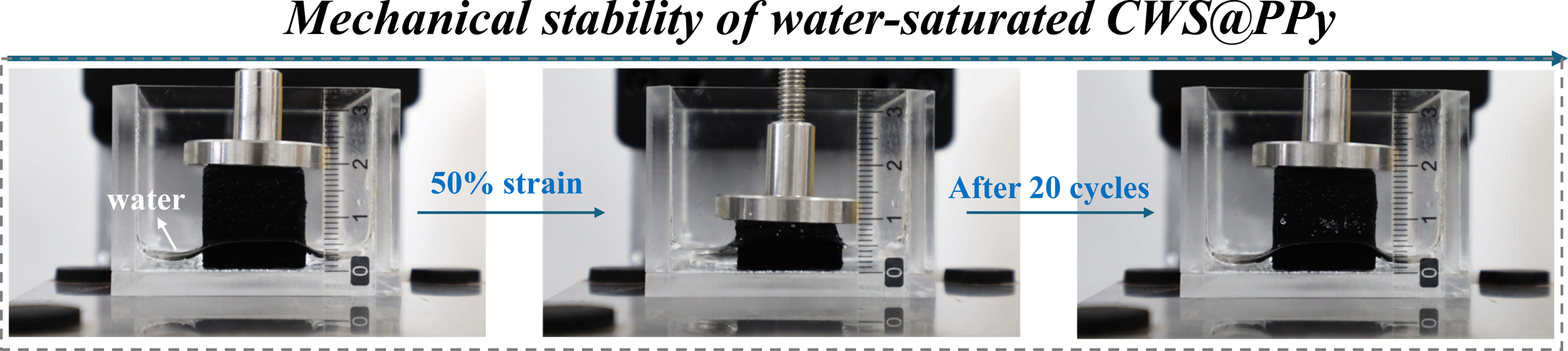


**Fig. S23** Photographs showing the mechanical stability of the water-saturated CWS@PPy


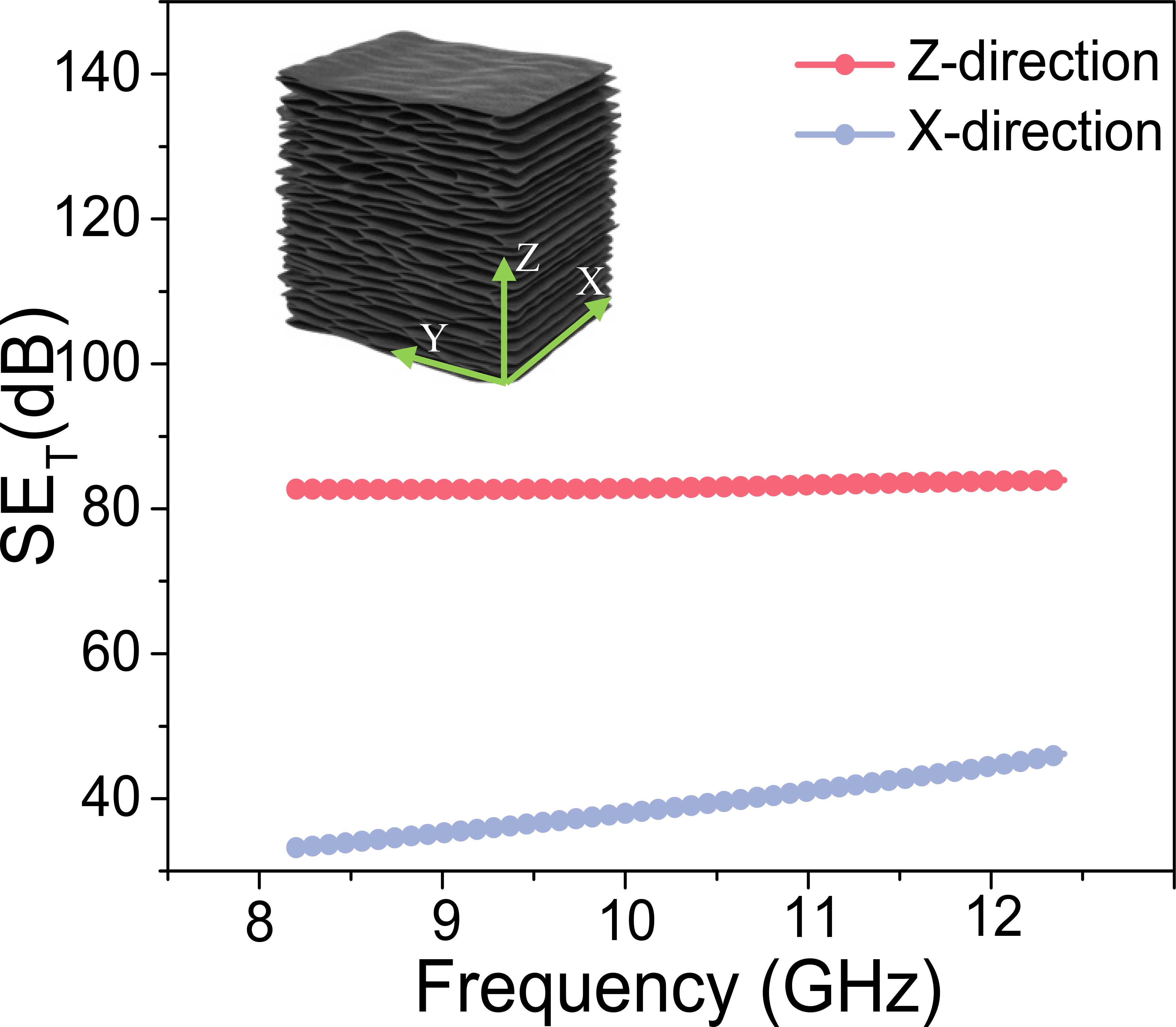


**Fig. S2****4** Anisotropic EMI shielding effectiveness of CWS@PPy


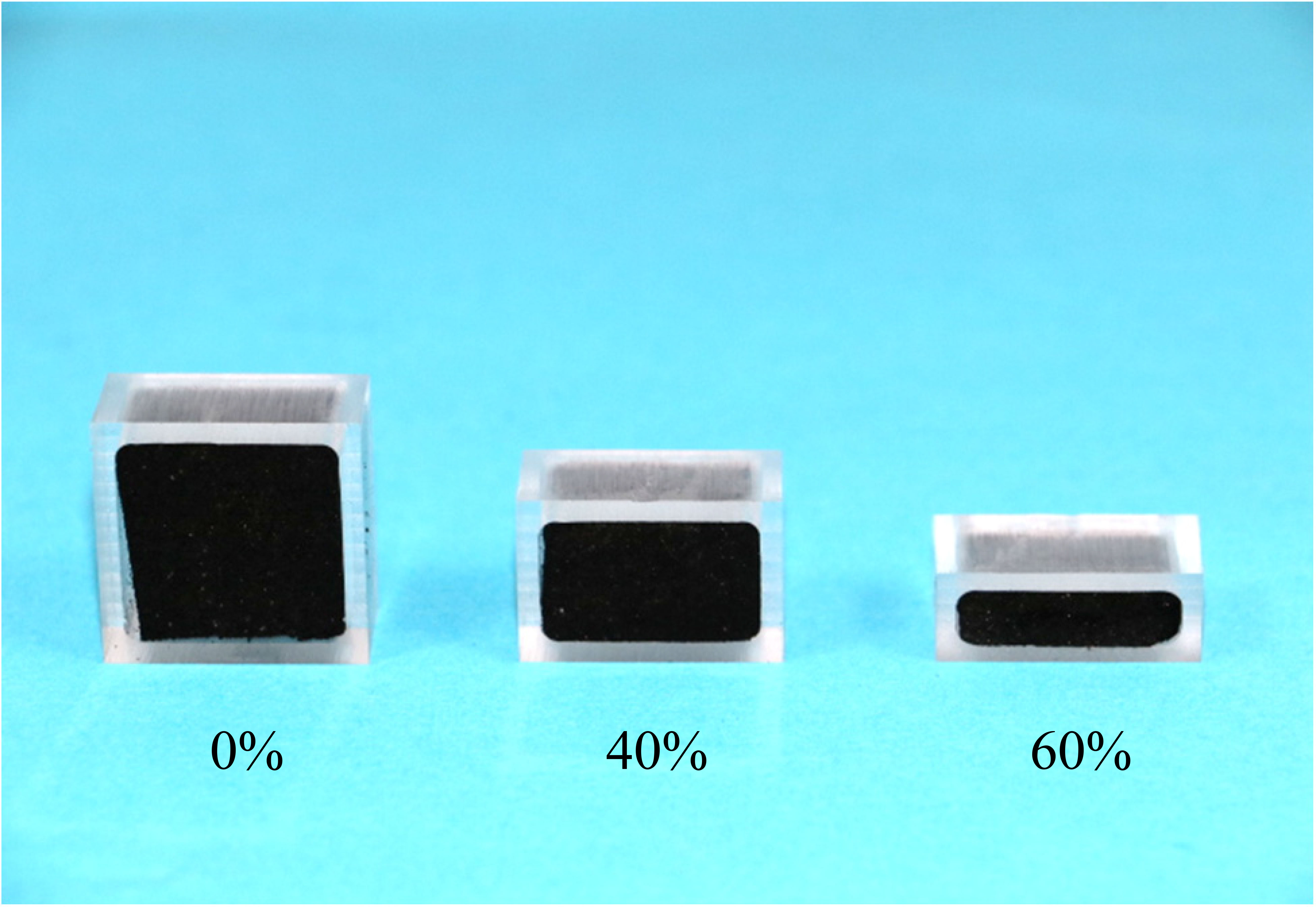


**Fig. S****25** Photograph of customized plastic mold with different sizes


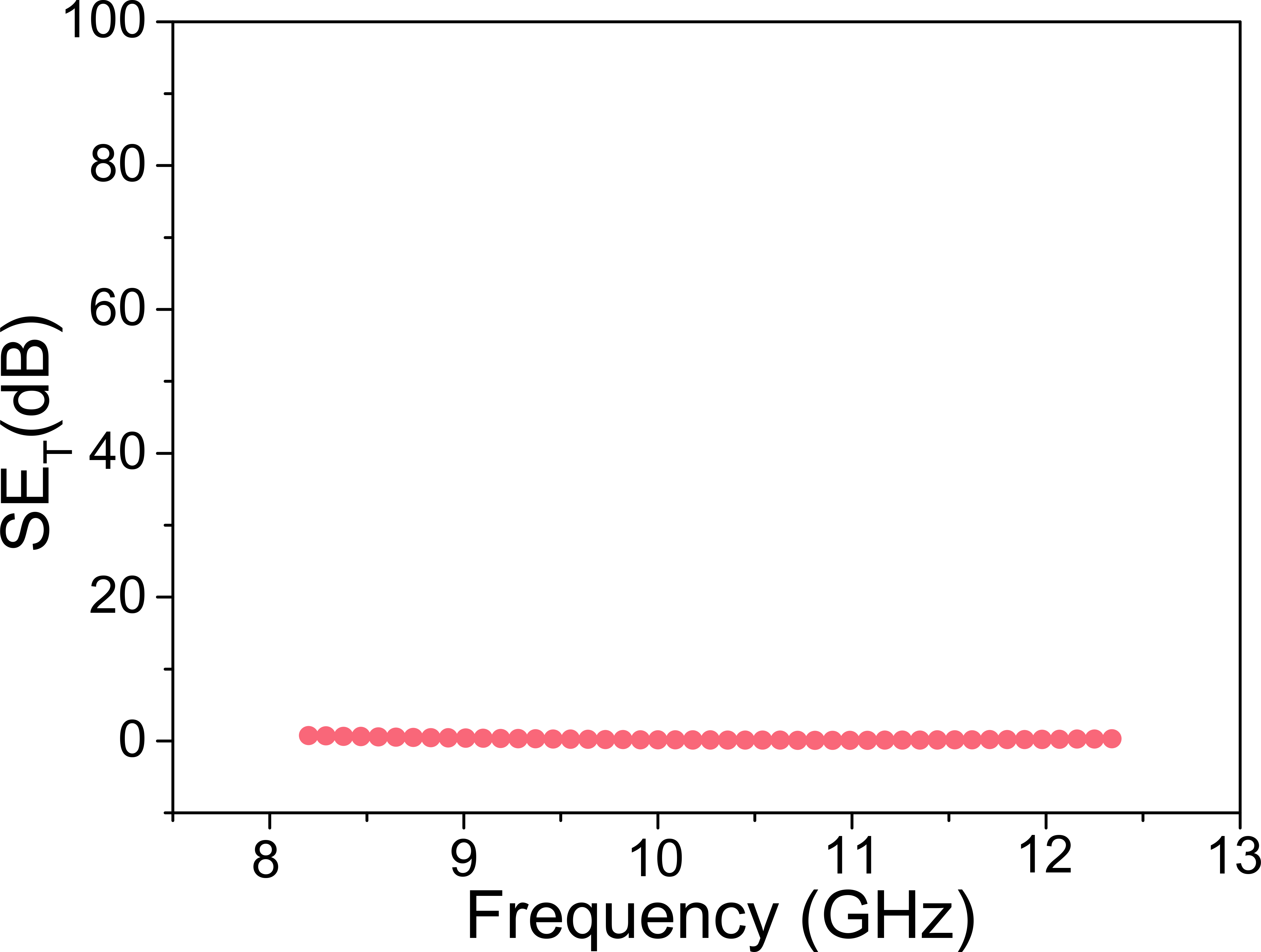


**Fig. S26** Electromagnetic shielding effectiveness of customized plastic mold


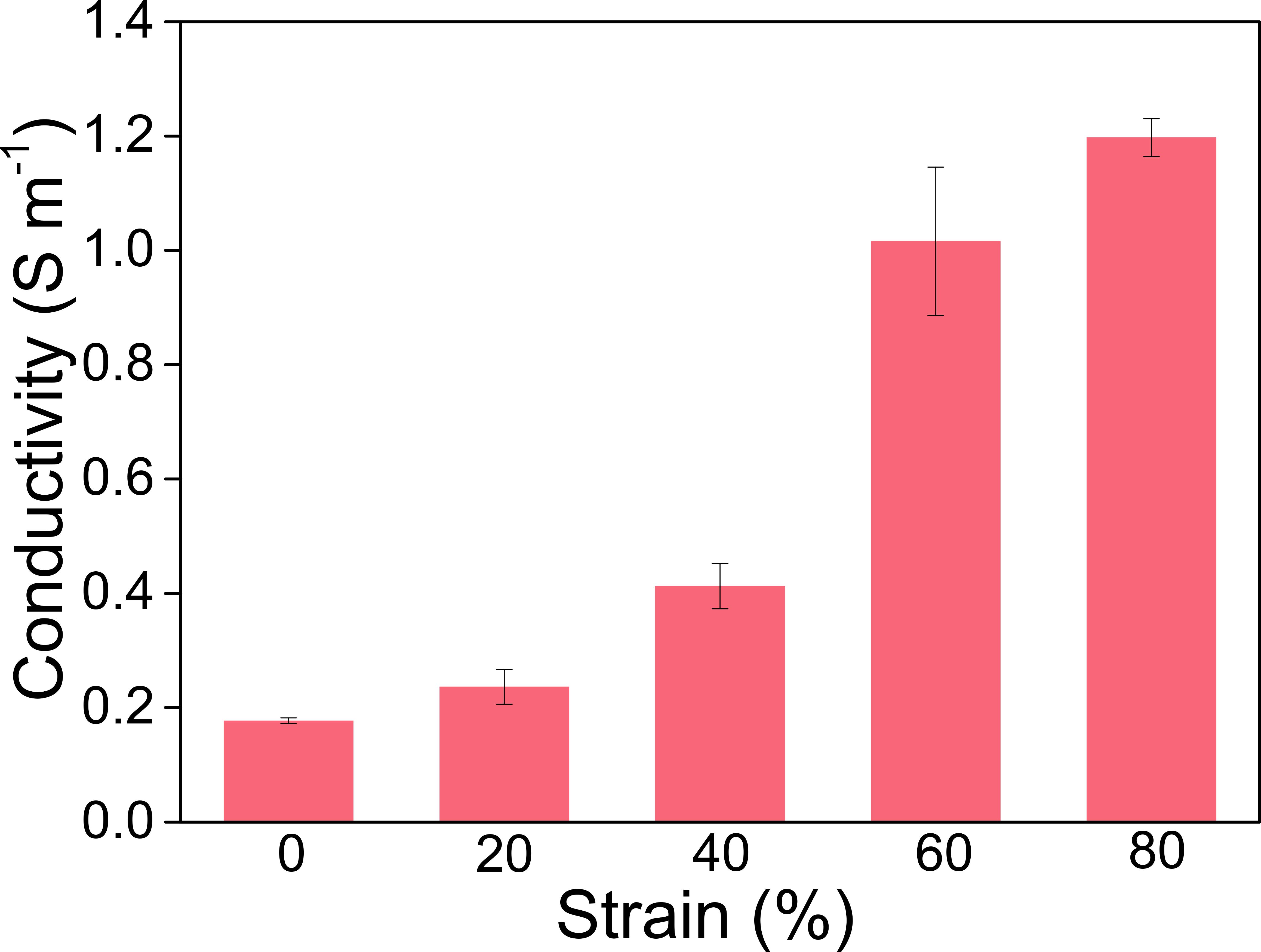


**Fig. S27** Electrical conductivity of CWS@PPy (17.51 wt%) at different strain


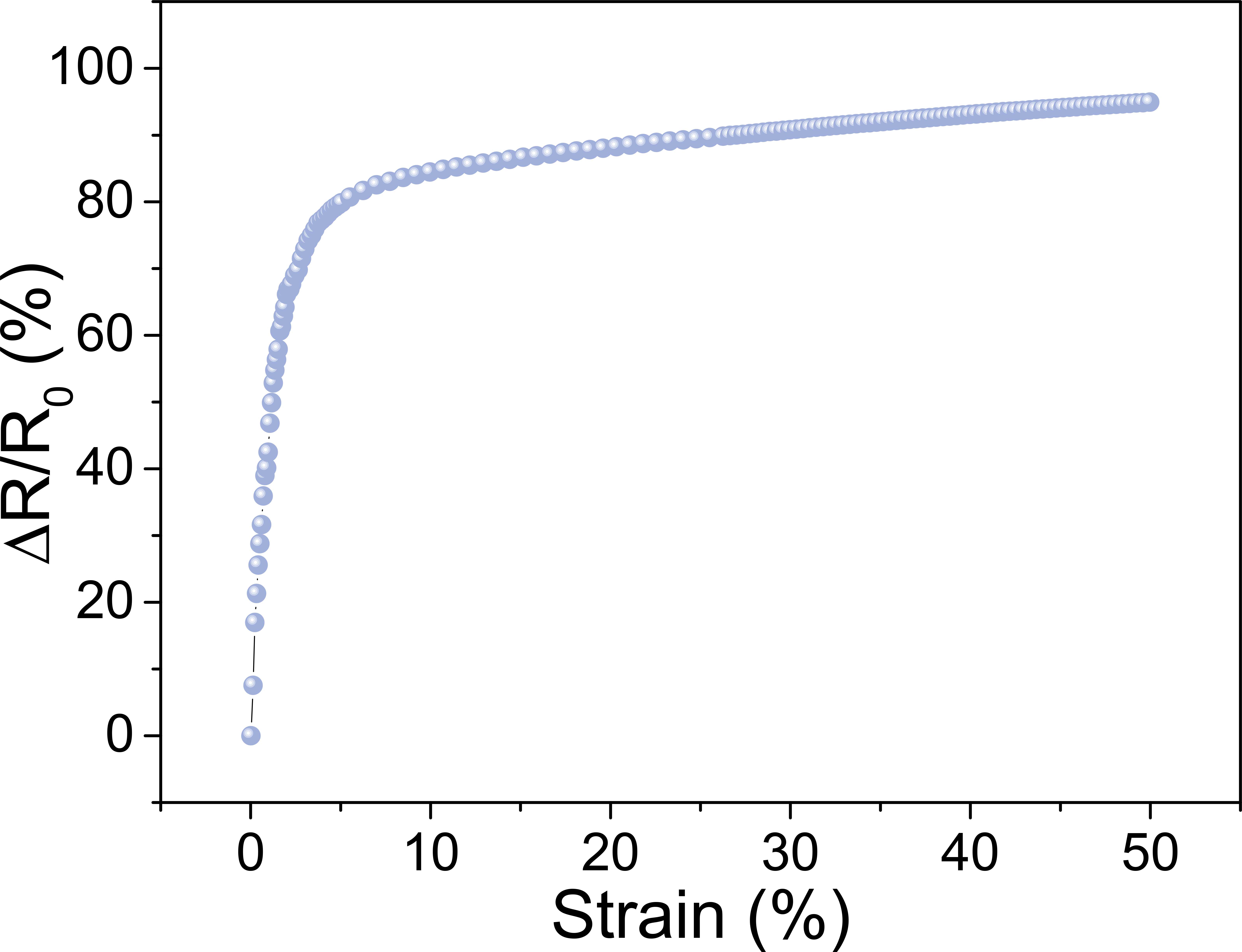


**Fig. S28** ΔR/R_0_ of the sensor as a function of strain


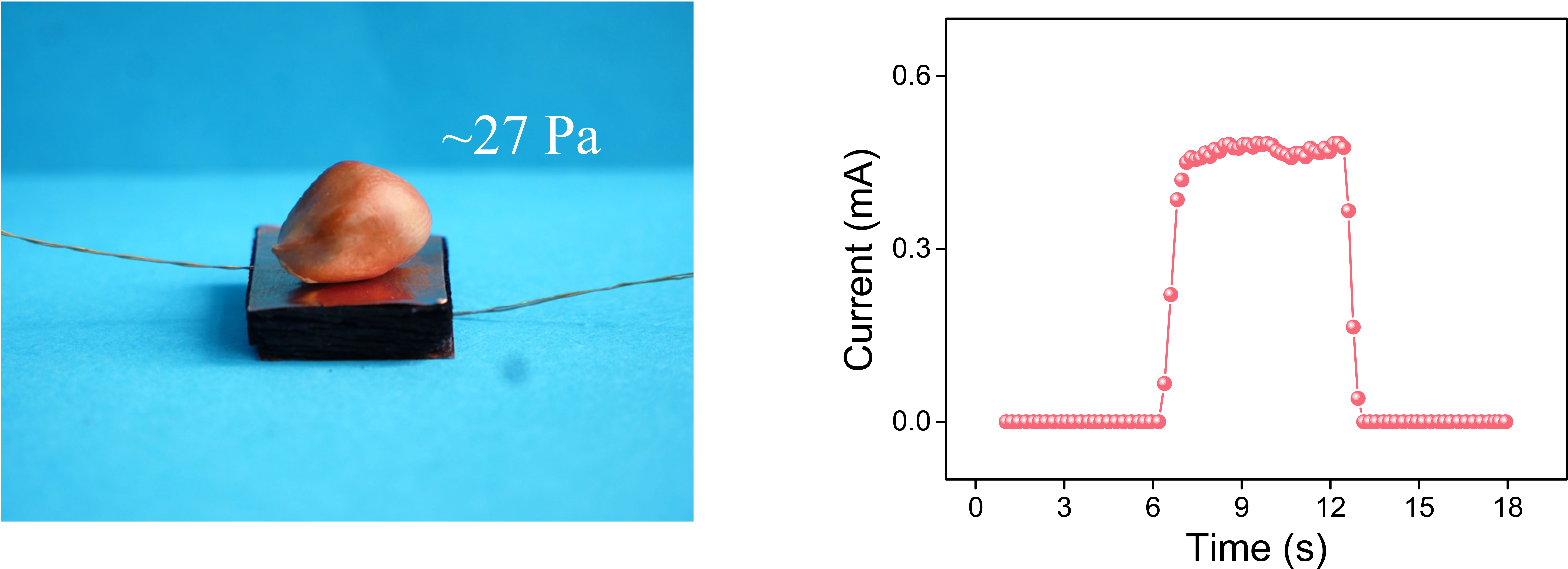


**Fig. S29** Current response of the CWS@PPy sensor loaded with an ultralight object (~27 Pa)


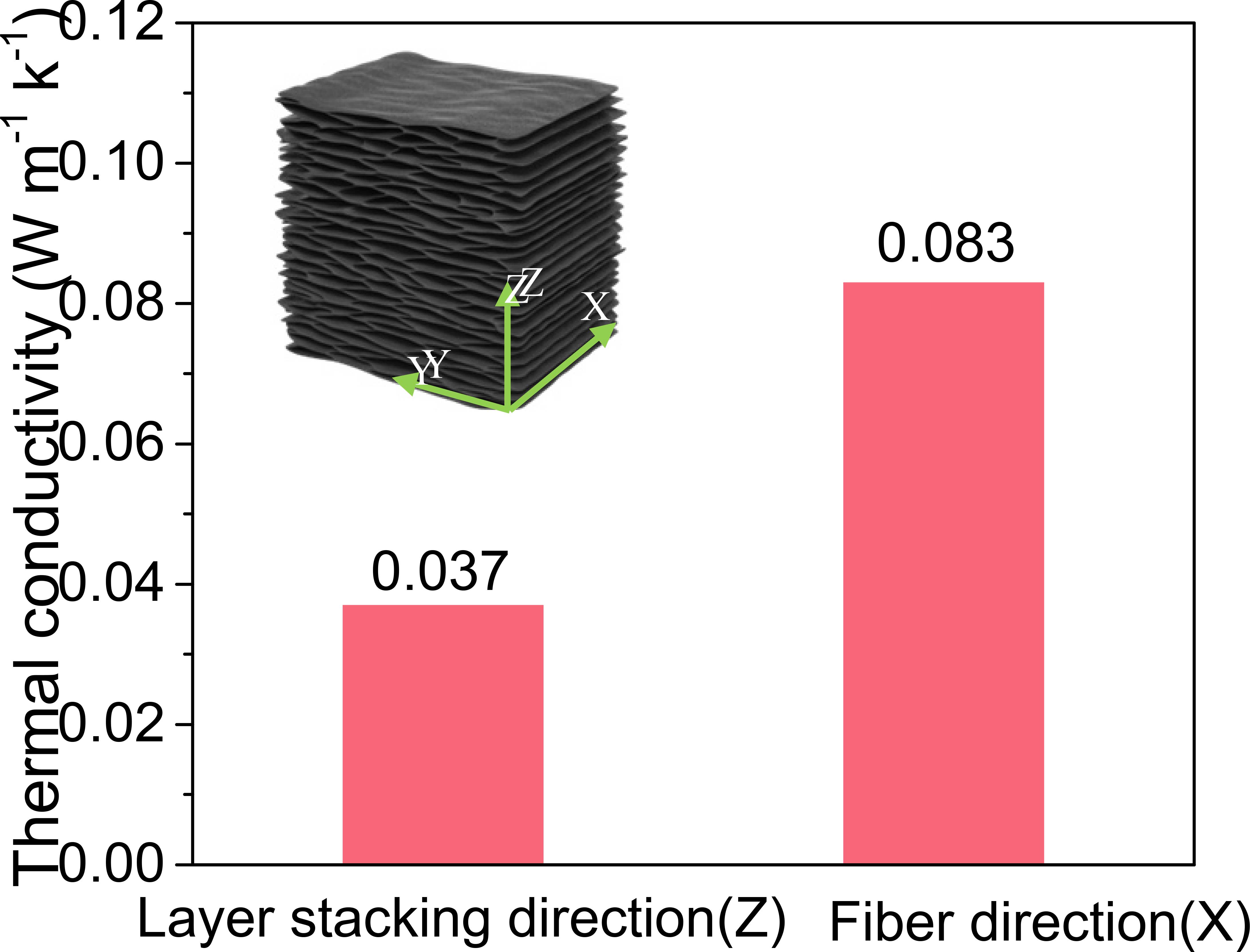


**Fig. S30** Anisotropic thermal conductivity of CWS@PPy


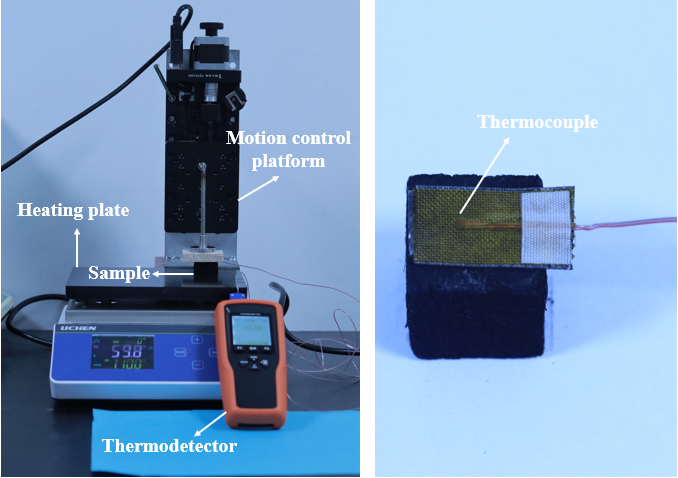


**Fig. S31** A homemade testing system for surface temperature measurement


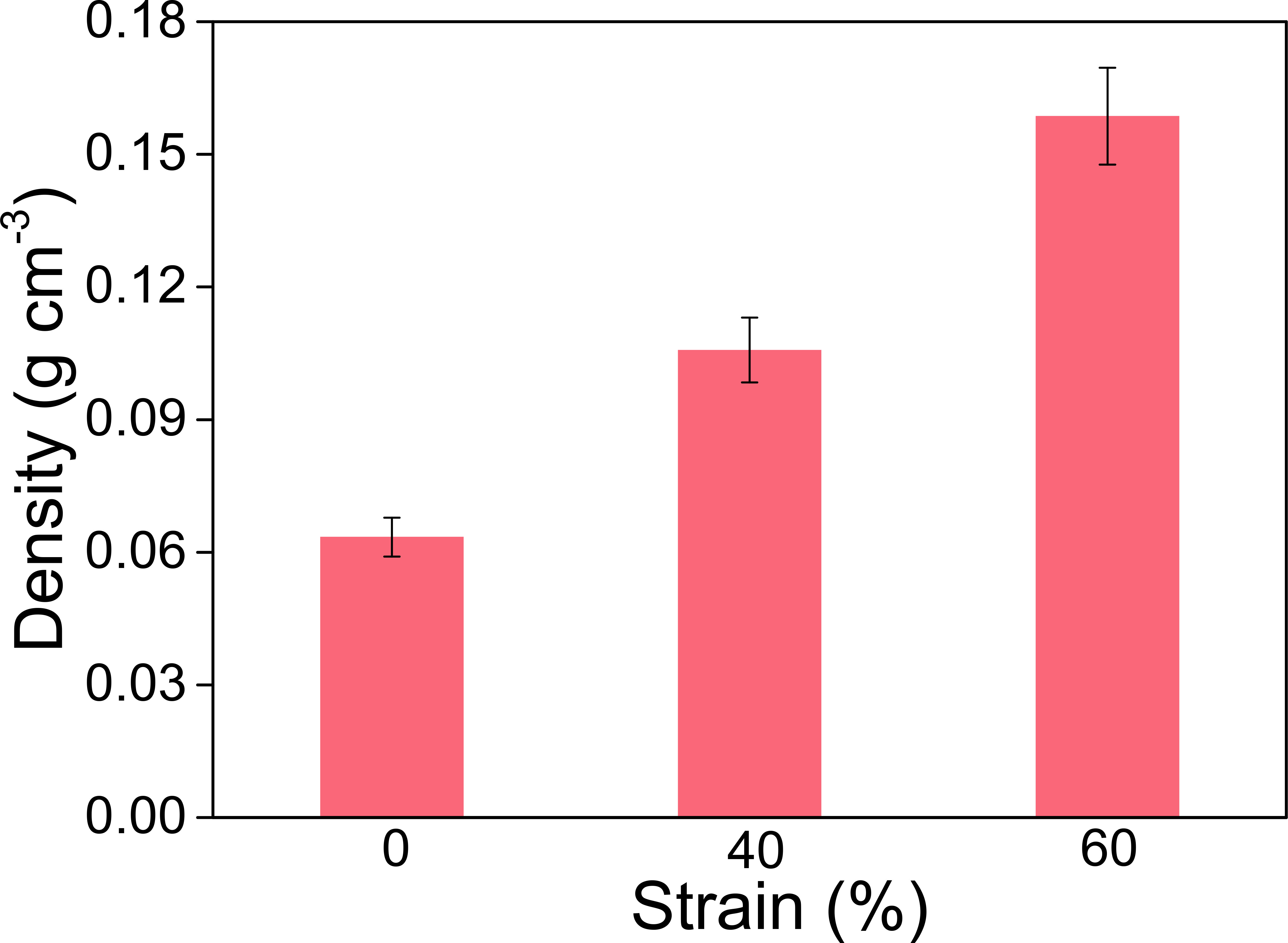


**Fig. S32** Density of CWS@PPy with different compressive strains





**Fig. S33** The integrated functions of CWS@PPy for innovative application

**Table S1** Comparison of the compressive fatigue resistance of CWS@PPy with those of the reported elastic aerogels and foams

| **Types** | **Materials** | **Strain** | | **Cycles** | | **Plastic deformation** | **Stress retention** | **Refs.** |
| --- | --- | --- | --- | --- | --- | --- | --- | --- |
| Biomass-based | KGM | | 50% | | 1000 | 4.3% | 75% | [S1] |
|  | CNF/PMSQ | | 60% | | 10 000 | 5.0% | 80% | [S2] |
|  | CNF/MOF | | 60% | | 800 | 8.0% | 70% | [S3] |
|  | CNF/CNT/MMT | | 50% | | 10 000 | 2.0% | 86.3% | [S4] |
|  | CNF/MOF | | 50% | | 1000 | 3.0% | 80.2% | [S5] |
| Synthetic polymer-based | PI/RGO | | 50% | | 2000 | 3.0% | 88% | [S6] |
|  | PI/PVP | | 50% | | 100 | 3.2% | 86.14% | [S7] |
| Carbon-based | RGO/PAA | | 50% | | 1000 | 3.5% | 82.0% | [S8] |
|  | RGO | | 70% | | 1000 | 10% | 80% | [S9] |
| Ceramic-based | SiO_2_/ | | 60% | | 1000 | 14.5% | 75% | [S10] |
|  | ZrO_2_/Al_2_O3 | | 60% | | 1000 | 12.5% | 70% | [S11] |
|  | **CWS@PPy** | | **40%** | | **10 000** | **3.5%** | **85%** | **This work** |

Note: Konjac glucomannan (KGM); Cellulose nanofibers (CNF); Polymethylsilsesquioxane (PMSQ); Metal-Organic Frameworks (MOF); Carbon nanotube (CNT); montmorillonite (MMT); Polyimide (PI); Polyvinylpyrrolidone (PVP); poly (amic acid) (PAA); Reduced graphene oxide (RGO).

**Table S2** Recovery speed of CWS@PPy and other previously reported materials

| **Materials** | **Recover speed** | **Refs.** |
| --- | --- | --- |
| GO-based monolith | 116.6 | [S12] |
| CS–GO based aerogel | 580.0 | [S13] |
| GO-based hybrid aerogel | 792.0 | [S14] |
| CNT-based aerogel | 847.0 | [S15] |
| Ceramic-based aerogel | 860.0 | [S16] |
| Polymer-based carbon aerogel | 860.0 | [S17] |
| GO-based aerogel | 1084.6 | [S18] |
| Ceramic-based sponge | 1233.0 | [S19] |
| **CWS@PPy** | **1111.0** | **This work** |

Note: Graphene oxide (GO); Chitosan (CS); Carbon nanotube (CNT)

**Table S3** Comparison of SSE value of CWS@PPy with other materials

| **Types** | **Materials** | | **Density**  **[g·cm^-3^]** | **Frequency**  **[GHz]** | **EMI SE**  **[dB]** | **SSE**  **[dB· cm^3^ ·g^-1^]** | **Refs.** |
| --- | --- | --- | --- | --- | --- | --- | --- |
| Wood-based | Carbonized Wood | 0.072 | | 8.2-12.4 | 77.0 | 1069.4 | [S20] |
|  | Delignified wood/PPy | 0.11 | | 8.2-12.4 | 22.99 | 209.0 | [S21] |
|  | Carbonized Wood/CNT | 0.541 | | 8.2-12.4 | 73.7 | 136.2 | [S22] |
|  | Wood/MXene | 0.108 | | 8.2-12.4 | 72.0 | 666.7 | [S23] |
|  | Carbonized Wood | 0.13 | | 8.2-12.4 | 60.463 | 465.1 | [S24] |
|  | Wood/MXene | 0.197 | | 8.2-12.4 | 71.3 | 361.9 | [S25] |
|  | Carbonized Wood | 0.48 | | 8.2-12.4 | 54.8 | 114.2 | [S26] |
| Polymer-based | Polyurethane/MXene | 0.063 | | 8.2-12.4 | 76.2 | 1209.5 | [S27] |
|  | polyimide foams | 0.091 | | 8.2-12.4 | 54.0 | 593.4 | [S28] |
|  | Polyimide/MXene/CNT | 0.152 | | 8.2-12.4 | 68.2 | 448.7 | [S29] |
|  | Polyimide/MXene | 0.0487 | | 8.2-12.4 | 62.5 | 1283.4 | [S30] |
|  | Polypropylene/CB | 0.12 | | 8.2-12.4 | 41.0 | 341.7 | [S31] |
|  | Polyurethane/CNT | 0.020 | | 8.2-12.4 | 23.0 | 1148.0 | [S32] |
| Cellulose-based | Cellulose/CNT | 0.095 | | 8.2-12.4 | 20.8 | 219 | [S33] |
|  | Cellulose/PANI | 0.1079 | | 8.2-12.4 | 85.4 | 791.2 | [S34] |
|  | Cellulose/GO | 0.0569 | | 8.2-12.4 | 58.4 | 1026.4 | [S35] |
|  | Nanocellulose/CNT | 0.075 | | 8.2-12.4 | 39.8 | 530.7 | [S36] |
|  | Nanocellulose/MXene | 0.1393 | | 8.2-12.4 | 110.0 | 789.7 | [S37] |
|  | Cellulose/CNT | 0.087 | | 8.2-12.4 | 40.2 | 461.95 | [S38] |
|  | Sugarcane | 0.112 | | 8.2-12.2 | 51.0 | 455.4 | [S39] |
|  | **CWS@PPy** | **0.06** | | **8.2-12.4** | **77.18** | **1286.3** | **This work** |

Note: Carbon nanotube (CNT), Graphene oxide (GO); carbon black (CB); Polyaniline (PANI);

**Table S4** Comparison of the maximum sensitivity of CWS@PPy sensor with various reported aerogel and foam-based sensors

| **Types** | **Materials** | **Sensitivity**  **[kPa^-1^]** | **Refs.** |
| --- | --- | --- | --- |
| Polymer-based | PI/MXene | 0.14 | [S40] |
|  | PDMS/Ni/EGaIn composite | 0.313 | [S41] |
|  | PU/RGO aerogel | 0.26 | [S42] |
|  | PU/CB aerogel | 0.068 | [S43] |
|  | Melamine/PANI/RGO composite | 0.152 | [S44] |
|  | PU/ANF/MXene | 0.46 | [S45] |
| Cellulose-based | Bamboo fiber/RGO sponge | 0.59 | [S46] |
|  | CNF/CB aerogel | 0.234 | [S47] |
|  | Bamboo cellulose nanofiber | 0.95 | [S48] |
|  | Cellulose carbon sponge | 1.04 | [S49] |
| Wood-based | Wood sponge/RGO | 0.32 | [S50] |
|  | Wood aerogel/PEDOT:PSS | 0.57 | [S51] |
|  | Wood sponge/CNT/ PEDOT:PSS | 1.05 | [S52] |
|  | Wood sponge/Grephene | 0.34 | [S53] |
|  | **CWS@PPy** | **0.72** | **This work** |

Note: Polyimide (PI); Polyurethane (PU); Reduced graphene oxide (RGO); Carbon black (CB); polyaniline (PANI); aramid fiber (ANF); Cellulose nanofiber (CNF); Carbon nanotube (CNT)

**Table S5** Comparison of thermal conductivity of CWS@PPy among reported aerogels and foams

| **Types** | **Materials** | **Temperature** | **Humidity** | **Thermal conductivity**  **[W m^-1^ K^-1^]** | **Refs.** |
| --- | --- | --- | --- | --- | --- |
|  | PBAT | RT | N/A | 0.0378 | [S54] |
| Polymer-based | PU | RT | N/A | 0.038 | [S55] |
|  | PI | RT | N/A | 0.059 | [S56] |
|  | CNF/GSA | RT | N/A | 0.03018 | [S57] |
|  | MFC/CNF/Al^3+^ | RT | N/A | 0.0403 | [S58] |
| Cellulose-based | CNF/PLA | RT | N/A | 0.041 | [S59] |
|  | CNF/G/Cu^2+^ | RT | N/A | 0.05 | [S60] |
|  | CNF/MH | RT | N/A | 0.056 | [S61] |
|  | Cel/BT | RT | 60% | 0.063 | [S62] |
|  | AL/PA | RT | 30% | 0.034 | [S63] |
|  | CNF/LGN | RT | N/A | 0.0383 | [S64] |
|  | LigSi | RT | 33% | 0.04 | [S65] |
| Biomass-based | Wastepaper | RT | N/A | 0.044 | [S66] |
|  | SiO2@CFs/SLS | RT | N/A | 0.046 | [S67] |
|  | SA/LS/PA | RT | N/A | 0.047 | [S68] |
|  | Pulp | RT | N/A | 0.068 | [S69] |
|  | Wood foams | RT | N/A | 0.028 | [S70] |
|  | Wood aerogel | RT | N/A | 0.028 | [S71] |
|  | Nanowood | RT | 20% | 0.03 | [S72] |
| Wood-based | Wood aerogel | RT | 50% | 0.037 | [S73] |
|  | Insulwood | RT | 50% | 0.038 | [S74] |
|  | Wood aerogel | RT | N/A | 0.094 | [S75] |
|  | **CWS@PPy** | **RT** | **25%** | **0.037** | **This work** |

Note: Poly (butylene adipate-co-terephthalate) (PBAT); Polyurethane (PU); Cellulose nanofiber (CNF); Glycerol succinic anhydride (GSA); Microfibrillated cellulose (MFC); Polylactic acid (PLA); Graphene (G); Magnesium hydroxide (MH); Cellulose (Cel); Bentonite (BT); Ammonium alginate (AL); Phytic acid (PA); Lignin (LGN); silica-mineralized lignin nanocomposite aerogel (LigSi); Sodium lignin sulfonate (SLS); Cellulose fibers (CFs); Sodium alginate (SA); Sodium lignosulfonate (LS);

**Supplementary References**

1. Y. Si, X. Wang, C. Yan, L. Yang, J. Yu et al., Ultralight biomass-derived carbonaceous nanofibrous aerogels with superelasticity and high pressure-sensitivity. Adv. Mater. **28**(43), 9512–9518 (2016). <https://doi.org/10.1002/adma.201603143>
2. J. Zhang, Y. Cheng, C. Xu, M. Gao, M. Zhu et al., Hierarchical interface engineering for advanced nanocellulosic hybrid aerogels with high compressibility and multifunctionality. Adv. Funct. Mater. **31**(19), 2009349 (2021). <https://doi.org/10.1002/adfm.202009349>
3. S. Zhou, V. Apostolopoulou-Kalkavoura, M.V. Tavares da Costa, L. Bergström, M. Strømme et al., Elastic aerogels of cellulose Nanofibers@Metal-organic frameworks for thermal insulation and fire retardancy. Nano-Micro Lett. **12**(1), 9 (2019). <https://doi.org/10.1007/s40820-019-0343-4>
4. T. Zhu, D. Wang, Y. Wang, F. Xu, J. Huang et al., Gelation-constrained freeze-casting fabrication of ultra-homogeneous nanocomposite aerogels with superelasticity and harsh environment tolerance. Adv. Funct. Mater. **35**(32), 2503693 (2025). <https://doi.org/10.1002/adfm.202503693>
5. J. Qiao, Q. Song, X. Zhang, S. Zhao, J. Liu et al., Enhancing interface connectivity for multifunctional magnetic carbon aerogels: an *in situ* growth strategy of metal-organic frameworks on cellulose nanofibrils. Adv. Sci. **11**(19), 2400403 (2024). <https://doi.org/10.1002/advs.202400403>
6. Y. Qin, Q. Peng, Y. Ding, Z. Lin, C. Wang et al., Lightweight, superelastic, and mechanically flexible graphene/polyimide nanocomposite foam for strain sensor application. ACS Nano **9**(9), 8933–8941 (2015). <https://doi.org/10.1021/acsnano.5b02781>
7. X. Zhao, K. Ruan, H. Qiu, X. Zhong, J. Gu, Fatigue-resistant polyimide aerogels with hierarchical cellular structure for broadband frequency sound absorption and thermal insulation. Adv. Compos. Hybrid Mater. **6**(5), 171 (2023). <https://doi.org/10.1007/s42114-023-00747-9>
8. Y. Zhang, P. Min, G. Yue, B. Niu, L. Li et al., Emulsion-based multiscale structural design realizes lightweight and superelastic graphene aerogels for electromagnetic interference shielding. Small **20**(48), 2405950 (2024). <https://doi.org/10.1002/smll.202405950>
9. X. Zhao, W. Yao, W. Gao, H. Chen, C. Gao, Wet-spun superelastic graphene aerogel millispheres with group effect. Adv. Mater. **29**(35), 1701482 (2017). <https://doi.org/10.1002/adma.201701482>
10. Y. Si, J. Yu, X. Tang, J. Ge, B. Ding, Ultralight nanofibre-assembled cellular aerogels with superelasticity and multifunctionality. Nat. Commun. **5**, 5802 (2014). <https://doi.org/10.1038/ncomms6802>
11. X. Zhang, F. Wang, L. Dou, X. Cheng, Y. Si et al., Ultrastrong, superelastic, and lamellar multiarch structured ZrO_2_–Al_2_O_3_ nanofibrous aerogels with high-temperature resistance over 1300 °C. ACS Nano **14**(11), 15616–15625 (2020). <https://doi.org/10.1021/acsnano.0c06423>
12. L. Qiu, J.Z. Liu, S.L.Y. Chang, Y. Wu, D. Li, Biomimetic superelastic graphene-based cellular monoliths. Nat. Commun. **3**, 1241 (2012). <https://doi.org/10.1038/ncomms2251>
13. H.-L. Gao, Y.-B. Zhu, L.-B. Mao, F.-C. Wang, X.-S. Luo et al., Super-elastic and fatigue resistant carbon material with lamellar multi-arch microstructure. Nat. Commun. **7**, 12920 (2016). <https://doi.org/10.1038/ncomms12920>
14. X. Wu, T. Liu, Y. Qiu, Z. Hou, C. Cai et al., Elastic yet strength triboelectric aerogel enabled by constructing a supramolecular system. Adv. Funct. Mater. **35**(11), 2417067 (2025). <https://doi.org/10.1002/adfm.202417067>
15. L. Zhuang, D. Lu, J. Zhang, P. Guo, L. Su et al., Highly cross-linked carbon tube aerogels with enhanced elasticity and fatigue resistance. Nat. Commun. **14**(1), 3178 (2023). <https://doi.org/10.1038/s41467-023-38664-6>
16. Y. Si, X. Wang, L. Dou, J. Yu, B. Ding, Ultralight and fire-resistant ceramic nanofibrous aerogels with temperature-invariant superelasticity. Sci. Adv. **4**(4), eaas8925 (2018). <https://doi.org/10.1126/sciadv.aas8925>
17. Z.-L. Yu, B. Qin, Z.-Y. Ma, J. Huang, S.-C. Li et al., Superelastic hard carbon nanofiber aerogels. Adv. Mater. **31**(23), e1900651 (2019). <https://doi.org/10.1002/adma.201900651>
18. L. Lv, P. Zhang, T. Xu, L. Qu, Ultrasensitive pressure sensor based on an ultralight sparkling graphene block. ACS Appl. Mater. Interfaces **9**(27), 22885–22892 (2017). <https://doi.org/10.1021/acsami.7b07153>
19. L. Li, C. Jia, Y. Liu, B. Fang, W. Zhu et al., Nanograin–glass dual-phasic, elasto-flexible, fatigue-tolerant, and heat-insulating ceramic sponges at large scales. Mater. Today **54**, 72–82 (2022). <https://doi.org/10.1016/j.mattod.2022.02.007>
20. Z. Dai, C. Hu, Y. Wei, W. Zhang, J. Xu et al., Highly anisotropic carbonized wood as electronic materials for electromagnetic interference shielding and thermal management. Adv. Electron. Mater. **9**(7), 2300162 (2023). <https://doi.org/10.1002/aelm.202300162>
21. W. Gan, C. Chen, M. Giroux, G. Zhong, M.M. Goyal et al., Conductive wood for high-performance structural electromagnetic interference shielding. Chem. Mater. **32**(12), 5280–5289 (2020). <https://doi.org/10.1021/acs.chemmater.0c01507>
22. M. Cheng, W. Ren, H. Li, X. Liu, S. Bandaru et al., Multiscale collaborative coupling of wood-derived porous carbon modified by three-dimensional conductive magnetic networks for electromagnetic interference shielding. Compos. Part B Eng. **224**, 109169 (2021). <https://doi.org/10.1016/j.compositesb.2021.109169>
23. M. Zhu, X. Yan, H. Xu, Y. Xu, L. Kong, Ultralight, compressible, and anisotropic MXene@Wood nanocomposite aerogel with excellent electromagnetic wave shielding and absorbing properties at different directions. Carbon **182**, 806–814 (2021). <https://doi.org/10.1016/j.carbon.2021.06.054>
24. Y. Yuan, X. Sun, M. Yang, F. Xu, Z. Lin et al., Stiff, thermally stable and highly anisotropic wood-derived carbon composite monoliths for electromagnetic interference shielding. ACS Appl. Mater. Interfaces **9**(25), 21371–21381 (2017). <https://doi.org/10.1021/acsami.7b04523>
25. C. Liang, H. Qiu, P. Song, X. Shi, J. Kong et al., Ultra-light MXene aerogel/wood-derived porous carbon composites with wall-like “mortar/brick” structures for electromagnetic interference shielding. Sci. Bull. **65**(8), 616–622 (2020). <https://doi.org/10.1016/j.scib.2020.02.009>
26. X. Liu, H. Liu, H. Xu, W. Xie, M. Li et al., Natural wood templated hierarchically cellular NbC/Pyrolytic carbon foams as Stiff, lightweight and High-Performance electromagnetic shielding materials. J. Colloid Interface Sci. **606**, 1543–1553 (2022). <https://doi.org/10.1016/j.jcis.2021.08.110>
27. E. Kim, H. Zhang, J.-H. Lee, H. Chen, H. Zhang et al., MXene/polyurethane auxetic composite foam for electromagnetic interference shielding and impact attenuation. Compos. Part A Appl. Sci. Manuf. **147**, 106430 (2021). <https://doi.org/10.1016/j.compositesa.2021.106430>
28. J. Li, Y. Ding, N. Yu, Q. Gao, X. Fan et al., Lightweight and stiff carbon foams derived from rigid thermosetting polyimide foam with superior electromagnetic interference shielding performance. Carbon **158**, 45–54 (2020). <https://doi.org/10.1016/j.carbon.2019.11.075>
29. T. Xue, Y. Yang, D. Yu, Q. Wali, Z. Wang et al., 3D printed integrated gradient-conductive MXene/CNT/polyimide aerogel frames for electromagnetic interference shielding with ultra-low reflection. Nano-Micro Lett. **15**(1), 45 (2023). <https://doi.org/10.1007/s40820-023-01017-5>
30. Z.-H. Zeng, N. Wu, J.-J. Wei, Y.-F. Yang, T.-T. Wu et al., Porous and ultra-flexible crosslinked MXene/polyimide composites for multifunctional electromagnetic interference shielding. Nano-Micro Lett. **14**(1), 59 (2022). <https://doi.org/10.1007/s40820-022-00800-0>
31. Y. Li, X. Lan, F. Wu, J. Liu, P. Huang et al., Steam-chest molding of polypropylene/carbon black composite foams as broadband EMI shields with high absorptivity. Compos. Commun. **22**, 100508 (2020). <https://doi.org/10.1016/j.coco.2020.100508>
32. Z. Zeng, H. Jin, M. Chen, W. Li, L. Zhou et al., Lightweight and anisotropic porous MWCNT/WPU composites for ultrahigh performance electromagnetic interference shielding. Adv. Funct. Mater. **26**(2), 303–310 (2016). <https://doi.org/10.1002/adfm.201503579>
33. H.-D. Huang, C.-Y. Liu, D. Zhou, X. Jiang, G.-J. Zhong et al., Cellulose composite aerogel for highly efficient electromagnetic interference shielding. J. Mater. Chem. A **3**(9), 4983–4991 (2015). <http://dx.doi.org/10.1039/C4TA05998K>
34. M. Chen, J. Zhu, K. Zhang, H. Zhou, Y. Gao et al., Carbon nanofiber/polyaniline composite aerogel with excellent electromagnetic interference shielding, low thermal conductivity, and extremely low heat release. Nano-Micro Lett. **17**(1), 80 (2024). <https://doi.org/10.1007/s40820-024-01583-2>
35. C. Wan, J. Li, Graphene oxide/cellulose aerogels nanocomposite: Preparation, pyrolysis, and application for electromagnetic interference shielding. Carbohydr. Polym. **150**, 172–179 (2016). <https://doi.org/10.1016/j.carbpol.2016.05.051>
36. G. Zhu, L. Giraldo Isaza, B. Huang, A. Dufresne, Multifunctional nanocellulose/carbon nanotube composite aerogels for high-efficiency electromagnetic interference shielding. ACS Sustainable Chem. Eng. **10**(7), 2397–2408 (2022). <https://doi.org/10.1021/acssuschemeng.1c07148>
37. L. Chen, T. Mai, X.-X. Ji, P.-L. Wang, M.-Y. Qi et al., 3D printing of customizable and lightweight multilayer MXene/nanocellulose architectures for tunable electromagnetic interference shielding *via* direct ink writing. Chem. Eng. J. **476**, 146652 (2023). <https://doi.org/10.1016/j.cej.2023.146652>
38. B. Haspulat Taymaz, V. Eskizeybek, Lightweight and sustainable recycled cellulose based hybrid aerogels with enhanced electromagnetic interference shielding. Cellulose **32**(5), 3335–3354 (2025). <https://doi.org/10.1007/s10570-025-06471-5>
39. Y.-Q. Li, Y.A. Samad, K. Polychronopoulou, K. Liao, Lightweight and highly conductive aerogel-like carbon from sugarcane with superior mechanical and EMI shielding properties. ACS Sustainable Chem. Eng. **3**(7), 1419–1427 (2015). <https://doi.org/10.1021/acssuschemeng.5b00340>
40. H. Liu, X. Chen, Y. Zheng, D. Zhang, Y. Zhao et al., Lightweight, superelastic, and hydrophobic polyimide nanofiber/MXene composite aerogel for wearable piezoresistive sensor and oil/water separation applications. Adv. Funct. Mater. **31**(13), 2008006 (2021). <https://doi.org/10.1002/adfm.202008006>
41. M. Stevens, G. Yun, T. Hasan, Porous conductive hybrid composite with superior pressure sensitivity and dynamic range. Adv. Funct. Mater. **34**(8), 2309347 (2024). <https://doi.org/10.1002/adfm.202309347>
42. H.-B. Yao, J. Ge, C.-F. Wang, X. Wang, W. Hu et al., A flexible and highly pressure-sensitive graphene–polyurethane sponge based on fractured microstructure design. Adv. Mater. **25**(46), 6692–6698 (2013). <https://doi.org/10.1002/adma.201303041>
43. X. Wu, Y. Han, X. Zhang, Z. Zhou, C. Lu, Large-area compliant, low-cost, and versatile pressure-sensing platform based on microcrack-designed carbon Black@Polyurethane sponge for human–machine interfacing. Adv. Funct. Mater. **26**(34), 6246–6256 (2016). <https://doi.org/10.1002/adfm.201601995>
44. G. Ge, Y. Cai, Q. Dong, Y. Zhang, J. Shao et al., A flexible pressure sensor based on rGO/polyaniline wrapped sponge with tunable sensitivity for human motion detection. Nanoscale **10**(21), 10033–10040 (2018). <http://dx.doi.org/10.1039/C8NR02813C>
45. K. Qian, J. Zhou, M. Miao, H. Wu, S. Thaiboonrod et al., Highly ordered thermoplastic polyurethane/aramid nanofiber conductive foams modulated by kevlar polyanion for piezoresistive sensing and electromagnetic interference shielding. Nanomicro Lett. **15**(1), 88 (2023). <https://doi.org/10.1007/s40820-023-01062-0>
46. Y. Lv, J. Wei, Z. Huang, Z. Zhang, S. Ding et al., Superelastic bamboo fiber-based spongy aerogel for flexible piezoresistive sensors with wide response range and high sensitivity. Chem. Eng. J. **488**, 151053 (2024). <https://doi.org/10.1016/j.cej.2024.151053>
47. Y. Zhang, P. Zhu, H. Sun, X. Sun, Y. Ye et al., Superelastic cellulose sub-micron fibers/carbon black aerogel for highly sensitive pressure sensing. Small **20**(13), 2310038 (2024). <https://doi.org/10.1002/smll.202310038>
48. Y. Lv, J. Wei, W. Wang, H. Deng, Z. Huang et al., Superelastic bamboo cellulose nanofiber based carbon aerogel with layered network microstructure for high sensitivity piezoresistive sensor. Int. J. Biol. Macromol. **295**, 139553 (2025). https://doi.org/10.1016/j.ijbiomac.2025.139553
49. W. Hu, D. Song, X. Shi, L. Zhou, Z. Zhao et al., Anisotropic electronic skin for neurofeedback. Adv. Funct. Mater. **34**(11), 2309359 (2024). <https://doi.org/10.1002/adfm.202309359>
50. H. Guan, X. Dai, L. Ni, J. Hu, X. Wang, Highly elastic and fatigue-resistant graphene-wrapped lamellar wood sponges for high-performance piezoresistive sensors. ACS Sustainable Chem. Eng. **9**(45), 15267–15277 (2021). <https://doi.org/10.1016/j.ijbiomac.2025.139553>
51. T. Liu, Z. Zhao, R. Liang, H. He, Y. Liu et al., Tough and elastic anisotropic triboelectric materials enabled by layer-by-layer assembly. Adv. Funct. Mater. **35**(26), 2500207 (2025). <https://doi.org/10.1002/adfm.202500207>
52. H. Zhang, Q. Zhang, J. Liang, B. Li, J. Zang et al., Pressure and temperature dual-parameter sensor based on natural wood for portable health-monitoring devices. ACS Sustainable Chem. Eng. **11**(45), 16194–16204 (2023). <https://doi.org/10.1021/acssuschemeng.3c04237>
53. G. Zhang, P. Li, X. Wang, Y. Xia, J. Yang, Flexible battery-free wireless sensor array based on functional gradient-structured wood for pressure and temperature monitoring. Adv. Funct. Mater. **33**(2), 2208900 (2023). <https://doi.org/10.1002/adfm.202208900>
54. Z. Wang, G. Wang, Z. Xu, C. Ma, G. Zhao, Pore-gradient, flexible, and fully-degradable foam with outstanding noise absorption and thermal insulation. Adv. Funct. Mater. **35**(37), 2504560 (2025). <https://doi.org/10.1002/adfm.202504560>
55. T.M. Nguyen-Ha, T.B. Nguyen, T.A. Nguyen, L.H. Pham, D.H. Nguyen et al., Novel high-performance sustainable polyurethane nanocomposite foams: Fire resistance, thermal stability, thermal conductivity, and mechanical properties. Chem. Eng. J. **474**, 145585 (2023). <https://doi.org/10.1016/j.cej.2023.145585>
56. X.-A. Li, Y. Zong, W. Li, J. Qian, Z. Qiang et al., Achieving ultrahigh transparency and superior mechanical properties in flexible polyimide nanofoams through CO_2_ foaming for thermal insulation. Adv. Funct. Mater. **34**(49), 2409498 (2024). <https://doi.org/10.1002/adfm.202409498>
57. Dinesh, B. Kumar, D.H. Pham, J. Kim, Strong, highly porous and sustainable nanocellulose foam made using bioderived hyperbranched crosslinker for thermal insulation and sound absorption. Carbohydr. Polym. **334**, 122016 (2024). <https://doi.org/10.1016/j.carbpol.2024.122016>
58. H. Sun, D. Zheng, Y. Zhu, P. Zhu, Y. Ye et al., Multiscale design for robust, thermal insulating, and flame self-extinguishing cellulose foam. Small **20**(12), 2306942 (2024). <https://doi.org/10.1002/smll.202306942>
59. Q. Ren, W. Li, S. Cui, W. Ma, X. Zhu et al., Improved thermal insulation and compressive property of bimodal poly (lactic acid)/cellulose nanocomposite foams. Carbohydr. Polym. **302**, 120419 (2023). <https://doi.org/10.1016/j.carbpol.2022.120419>
60. C. Chen, Y. Zhou, W. Xie, T. Meng, X. Zhao et al., Lightweight, thermally insulating, fire-proof graphite-cellulose foam. Adv. Funct. Mater. **33**(6), 2204219 (2023). <https://doi.org/10.1002/adfm.202204219>
61. Y. Han, X. Zhang, X. Wu, C. Lu, Flame retardant, heat insulating cellulose aerogels from waste cotton fabrics by *in situ* formation of magnesium hydroxide nanoparticles in cellulose gel nanostructures. ACS Sustainable Chem. Eng. **3**(8), 1853–1859 (2015). <https://doi.org/10.1021/acssuschemeng.5b00438>
62. L. Chen, S. Wang, S. Wang, C. Chen, L. Qi et al., Scalable production of biodegradable, recyclable, sustainable cellulose–mineral foams *via* coordination interaction assisted ambient drying. ACS Nano **16**(10), 16414–16425 (2022). <https://doi.org/10.1021/acsnano.2c05635>
63. M. Cao, B.-W. Liu, L. Zhang, Z.-C. Peng, Y.-Y. Zhang et al., Fully biomass-based aerogels with ultrahigh mechanical modulus, enhanced flame retardancy, and great thermal insulation applications. Compos. Part B Eng. **225**, 109309 (2021). <https://doi.org/10.1016/j.compositesb.2021.109309>
64. H. Wang, J. Kim, Development of lightweight, high-strength, and highly porous ligno-nanocellulosic foam with excellent antioxidant and insulation properties. Carbohydr. Polym. **326**, 121616 (2024). <https://doi.org/10.1016/j.carbpol.2023.121616>
65. Q. Fan, R. Ou, X. Hao, Q. Deng, Z. Liu et al., Water-induced self-assembly and *in situ* mineralization within plant phenolic glycol-gel toward ultrastrong and multifunctional thermal insulating aerogels. ACS Nano **16**(6), 9062–9076 (2022). <https://doi.org/10.1021/acsnano.2c00755>
66. B. Zhang, W. Tao, Z. Ren, S. Yue, J. Gou, Biodegradable wastepaper-based foam with ultrahigh energy-absorbing, excellent thermal insulation, and outstanding cushioning properties. ACS Sustainable Chem. Eng. **11**(49), 17543–17551 (2023). <https://doi.org/10.1021/acssuschemeng.3c06230>
67. H. Dong, S. Wei, W. Chen, B. Lu, Z. Cai et al., Bioinspired lignocellulose foam: exceptional toughness and thermal insulation. ACS Nano **19**(12), 11712–11727 (2025). <https://doi.org/10.1021/acsnano.4c11945>
68. Q. Cen, S. Chen, D. Yang, D. Zheng, X. Qiu, Full bio-based aerogel incorporating lignin for excellent flame retardancy, mechanical resistance, and thermal insulation. ACS Sustainable Chem. Eng. **11**(11), 4473–4484 (2023). <https://doi.org/10.1021/acssuschemeng.2c07652>
69. M. Wu, G. Yu, W. Chen, S. Dong, Y. Wang et al., A pulp foam with highly improved physical strength, fire-resistance and antibiosis by incorporation of chitosan and CPAM. Carbohydr. Polym. **278**, 118963 (2022). <https://doi.org/10.1016/j.carbpol.2021.118963>
70. X. Shi, R. Bi, Z. Wan, F. Jiang, O.J. Rojas, Solid wood modification toward anisotropic elastic and insulative foam-like materials. ACS Nano **18**(11), 7959–7971 (2024). <https://doi.org/10.1021/acsnano.3c10650>
71. J. Song, C. Chen, Z. Yang, Y. Kuang, T. Li et al., Highly compressible, anisotropic aerogel with aligned cellulose nanofibers. ACS Nano **12**(1), 140–147 (2018). <https://doi.org/10.1021/acsnano.7b04246>
72. T. Li, J. Song, X. Zhao, Z. Yang, G. Pastel et al., Anisotropic, lightweight, strong, and super thermally insulating nanowood with naturally aligned nanocellulose. Sci. Adv. **4**(3), eaar3724 (2018). <https://doi.org/10.1126/sciadv.aar3724>
73. J. Garemark, J.E. Perea-Buceta, D. Rico del Cerro, S. Hall, B. Berke et al., Nanostructurally controllable strong wood aerogel toward efficient thermal insulation. ACS Appl. Mater. Interfaces **14**(21), 24697–24707 (2022). <https://doi.org/10.1021/acsami.2c04584>
74. X. Zhao, Y. Liu, L. Zhao, A. Yazdkhasti, Y. Mao et al., A scalable high-porosity wood for sound absorption and thermal insulation. Nat. Sustain. **6**(3), 306–315 (2023). <https://doi.org/10.1038/s41893-022-01035-y>
75. Z. Liu, T. Liu, H. Dong, B. Yang, X. Li et al., Diatom-inspired nanoscale heterogeneous assembly strategy for constructing thermal insulating wood-based aerogels with exceptional strength, resilience, degradability, and flame retardancy. ACS Nano **19**(7), 6826–6839 (2025). <https://doi.org/10.1021/acsnano.4c11549>
